# A Two-Step Synthesis of Unprotected 3-Aminoindoles via Post Functionalization with Nitrostyrene

**DOI:** 10.3390/molecules28093657

**Published:** 2023-04-23

**Authors:** Nicolai A. Aksenov, Nikolai A. Arutiunov, Igor A. Kurenkov, Vladimir V. Malyuga, Dmitrii A. Aksenov, Daria S. Momotova, Anna M. Zatsepilina, Elizaveta A. Chukanova, Alexander V. Leontiev, Alexander V. Aksenov

**Affiliations:** Department of Chemistry, North Caucasus Federal University, 1a Pushkin St., 355009 Stavropol, Russia; naarutiunov@ncfu.ru (N.A.A.); kurenkman@icloud.com (I.A.K.); vmaliuga@ncfu.ru (V.V.M.); daksenov@ncfu.ru (D.A.A.); momotova.s52@gmail.com (D.S.M.); ann.zazaza.2002@gmail.com (A.M.Z.); elizavetac751@gmail.com (E.A.C.); alvlleontev@ncfu.ru (A.V.L.)

**Keywords:** C3-amination of indoles, cascade transformations, phenylacetonitrile extrusion

## Abstract

A novel, low-cost method for the preparation of not easily accessible free 3-aminoindoles has been developed. This approach is based on a well-established reaction between indoles and nitrostyrene in the presence of phosphorous acid, which results in the formation of 4′-phenyl-4′H-spiro[indole-3,5′-isoxazoles]. The latter could be transformed to corresponding aminated indoles by reaction with hydrazine hydrate in good or excellent yields upon microwave-assisted heating.

## 1. Introduction

3-aminoindolic motif commonly occurs in many natural and artificial substances demonstrating a wide range of biological activities [1,2,3,4,5]. Ideally, to install this fragment into the target molecules, one would need a convenient, broadly applicable synthetic pathway to free, 3-aminated indoles as corresponding precursors or building blocks. However, the instability of the unprotected, electron-rich 3-aminoindoles, which are sensitive to light and air and tend to undergo oxidative dimerization and/or other types of decomposition reactions [6,7,8] is the main reason why such a method is still yet to be found. Until then, most of the reported synthesizes toward 3-aminoindoles are usually dealt with relatively stable electron-pour-deactivated derivatives [9,10,11,12] or rely on capping the in situ generated amino group with suitable protective groups [13,14,15,16,17]. Up to date, there are just a few published procedures where unprotected 3-aminoindoles were isolated and characterized [7,8,18].

**Scheme 1:**
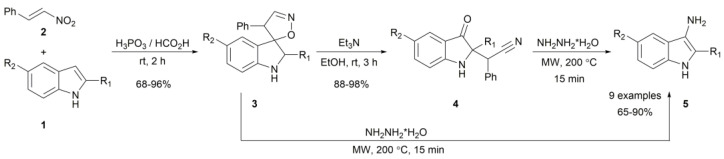
Two-step C3-amination of 2-arylindoles with nitrostyrene and hydrazine hydrate.

On the other hand, common approaches to 3-aminoindole derivatives can be roughly divided into two kinds: non-indolic methods, which build up the aminoindole skeleton from scratch via the Fisher [19,20] or similar multicomponent annulation reactions [7,8,9,10,11,12,14,21,22,23,24] and post-functionalization procedures based on the corresponding 3-substituted indoles. As for the latter, the major strategies for introducing an amino group at the C3 indole position have remained the nitration [2,13,15,17,25,26] or azidation [18] reactions followed by a reduction to the free amine. The synthesis starts from the corresponding 3-indolecarboxylic acids by a two-step sequence involving the Curtius rearrangement [16,27], palladium-catalyzed amination of indole halides [28], as well as a number of some recent direct C-H amination methods [29,30,31,32,33,34], have been also reported. However, these transformations are mostly multistep processes that require subsequent protection–deprotection or functional group interconversion steps and often suffer from limited scope and efficacy. In turn, herein, we would like to present a novel two-step method for the preparation of unprotected 2-aryl-3-aminoindoles **5** directly from the corresponding 2-aryl indoles **1** and nitrostyrene **2** via intermedial spirocyclic isoxazoles **3** or indolinone **4** (Scheme 1). This approach provides a straightforward synthetic route to otherwise not easily accessible free 3-aminoindoles.

## 2. Results

**Scheme 2:**
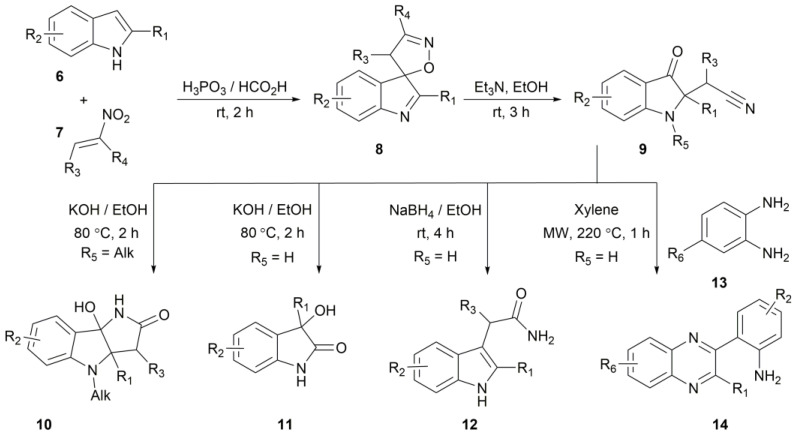
Some transformations based on 2-(3-oxoindolin-2-yl)-2-arylacetonitriles **9**.

A few years ago, we have discovered [35,36] a somewhat unusual reaction between indoles **6** and nitrostyrenes **7**, where the latter act as 1,4-dipoles in the presence of phosphorous acid to give diastereomerically pure spiro-2-indolinone isoxazoles **8** in good to excellent yields (Scheme 2). Subsequent treatment with a mild acid or base leads to 2-(3-oxoindolin-2-yl)-2-arylacetonitriles **9** [37,38], which are due to the presence of versatile cyano and carbonyl functional groups could serve as a good synthetic platform for carrying out many other useful transformations.

Thus, so far, we have shown that upon the action of KOH in refluxing ethanol, the N-alkyl indolinones **9** formed pyrroloindoles **10**, while NH derivatives under the same conditions demonstrated the unexpected extrusion of arylacetonitrile molecule, which eventually result in the formation of hydroxyindolinones **11** [39]. The reduction of **8** or **9** with sodium borohydride has proved to be an efficient way of preparation of corresponding indolylacetamides **12** [40], and a reaction of 2-(3-oxoindolin-2-yl)-2-arylacetonitriles **9** with benzene-1,2-diamines **13** furnished quinoxalines **14** in high-yields [41].

**Scheme 3:**
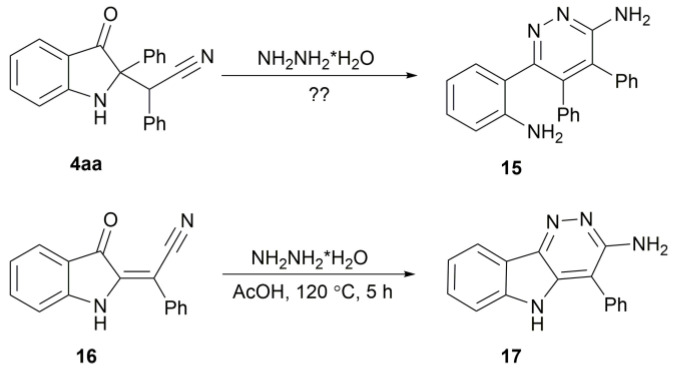
The speculative approach to aminopyridazine **15**.

It should be noticed that the latter transformation is accompanied by loss of phenylacetonitrile molecule, so, at some point, we speculated about using hydrazine hydrate instead of *o*-phenylenediamine derivatives to avoid it. In this case, starting, for instance, from indolinone **4aa**, one would expect aminopyridazine **15** as a product analogously to the results of the work [42] (Scheme 3). The authors of that work used the structurally similar substrate **16** to obtain pyridazinoindole **17** by cyclocondensation, the former with hydrazine hydrate in boiling acetic acid.

**Scheme 4:**
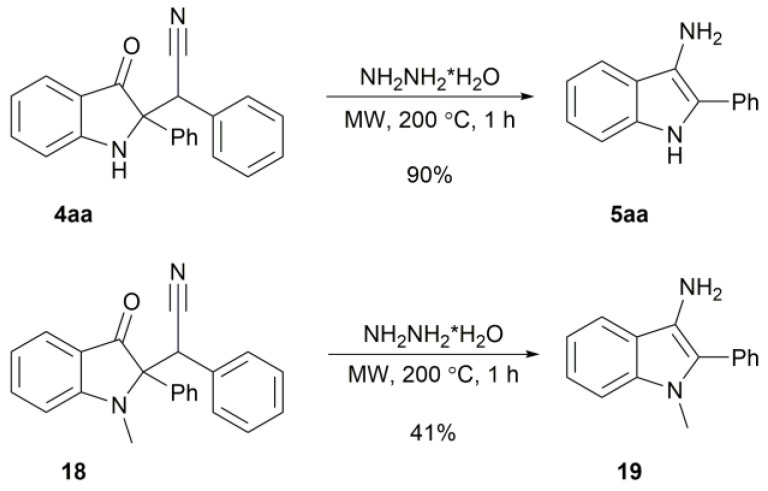
The unexpected formation of 3-aminoindoles.

Keeping that in mind, firstly, we tried to reflux **4aa** in hydrazine hydrate, only to get the starting material back unchanged. In the attempt to force the reaction, microwave-assisted heating at 200 °C for 1 h was applied, and this time, the conversion of **4aa** did occur, affording, to our surprise, 3-amino-2-phenylindole **5aa** as the only isolable product (Scheme 4). In turn, the *N*-methylated indolinone **18** under the same conditions furnished the corresponding N-Me 3-aminoindole **19**, although in a rather modest 41% yield.

Apparently, as opposed to our initial hypothesis, the use of hydrazine hydrate (Entry 9) with **4aa** does not lead to the target aminopyridazine **15** nor other hydrazines (Entries 3–8), and nitrogen binucleophiles (Entries 1,2,4–9) lead each time to the 3-aminoindole **5aa** (Table 1).

**Scheme 5:**
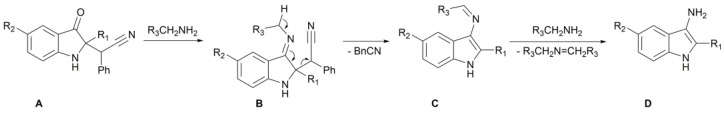
Proposed mechanism of 3-aminoindoles formation with R_3_CH_2_NH_2_-type nucleophiles.

To come up with a plausible mechanism, one had to accommodate all those observations above, namely the formation of the 3-aminoindole **5aa** with both R_3_CH_2_NH_2_ (Entries 1,2) and hydrazine (Entries 3–9) derivatives while lacking any expected products in the case of hydroxylamine (Entry 10). We speculate (Scheme 5) that for the amine-bearing α-CH_2_ group (such as ethylene or 1,2-propylenediamines), the condensation of the latter with the starting indolinone **A** leads to the corresponding imine **B**. The following proton abstraction from the *N*-alkylimino fragment, accompanied by simultaneous benzyl cyanide loss, gives a new, aromatization-driven imine **C**. Then, due to an excess of R_3_CH_2_NH_2_, a recondensation occurs, resulting in the target aminated indole **D**. In favor of this mechanism, a key feature of which is the presence of α-CH protons, speaks to our previous finding [41], where a reaction of (3-oxoindolin-2-yl)acetonitriles **A** with 1,2-phenylenediamines ends up in quinoxalines **14** (Scheme 2). If anything like that were to happen in the case of 1,2-diamines (Entries 1,2), then the formation of the corresponding dihydroquinaxolines was expected but did not occur.

**Scheme 6:**
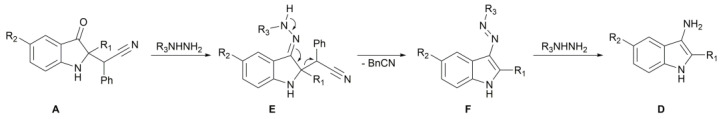
Proposed mechanism of 3-aminoindoles formation with R_3_NHNH_2_-type nucleophiles.

Meanwhile, in the case of hydrazine derivatives (Entries 3–9), supposedly, the reaction takes a slightly different pathway (Scheme 6). First of all, like with imine **B** (Scheme 5), the formation of hydrazone **E** should occur. The following extrusion of a phenylacetonitrile molecule would lead to the azo-heteroarene **F**. As shown previously, the -N=N- bond undergoes a reductive cleavage to the corresponding anilines upon the action of (among other reductants) hydrazine hydrate in the presence of, for instance, Raney Ni [43], aluminum powder [44], or even without any catalyst by simple heating in ethanol [45]. We assume that something similar takes place in our case resulting eventually in the target 3-aminoindole **D**.

**Scheme 7:**
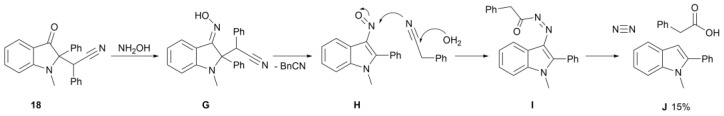
Plausible mechanism of formation of 1-methyl-2-phenylindole **J**.

Lastly, in the reaction with hydroxylamine (Entry 10), no meaningful products were isolated. However, the *N*-methyl derivative **18**, under the same conditions, unexpectedly gave 1-methyl-2-phenylindole **J**, although with a low 15% yield (Scheme 7). Most likely, the corresponding oxime **G**, after the loss of the BnCN molecule, became nitrosoindole **H**. Further hydrolysis of benzyl cyanide and its condensation with the nitroso group of indole **H** produced the azo compound **I**, which in turn got reduced in some Wolff–Kishner-type reaction to form the 1-methyl-2-phenylindole **J**.

**Scheme 8:**
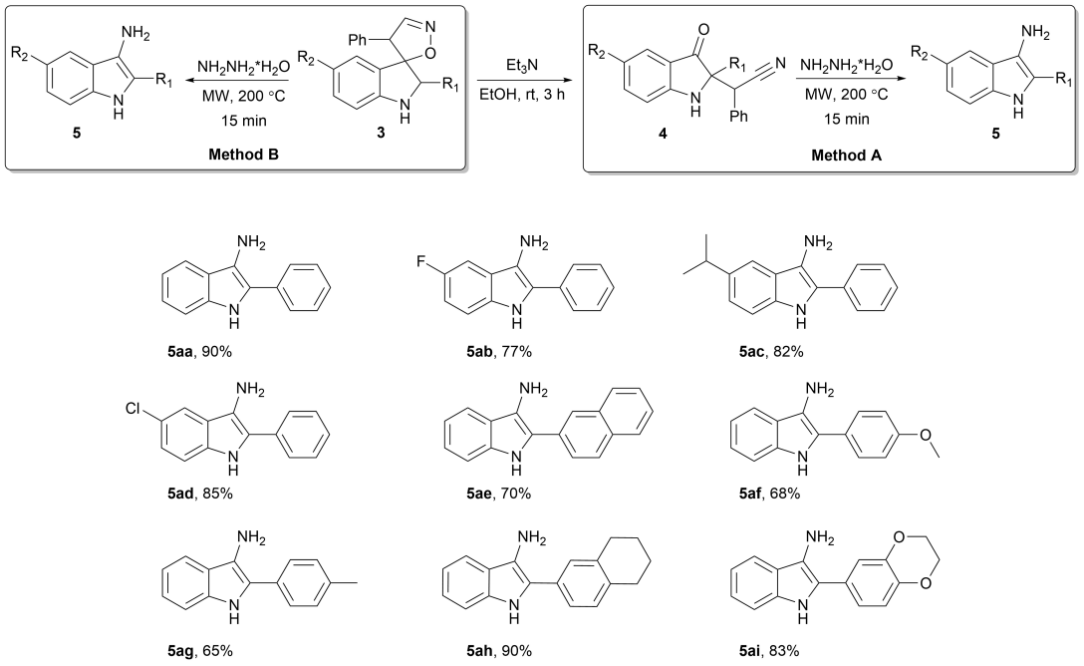
Unprotected 2-aryl-3-aminoindoles prepared by described herein procedure.

Next, we evaluated the scope and limitations of the described procedure (Entry 9). For that, a series of indolinones **4** bearing various aryl substituents R_1_ was introduced into the reaction with hydrazine hydrate under the chosen conditions (Method A). As seen in Scheme 8, all these substrates reacted smoothly, producing the corresponding products **5aa**–**5ag** in good to high yields. The presence of alkyl or fluorine substitutes at C-5 in the indoline core did not affect the reaction performance, and the target 5-substituted 3-aminoindoles **5ab**, **5ac**, and **5ad** were also obtained in high yields (Scheme 8). Remarkably, the direct conversion of spiranes **3** into aminoindoles **5** (Method B) is also possible, giving yields comparable to those obtained via Method A.

**Scheme 9:**
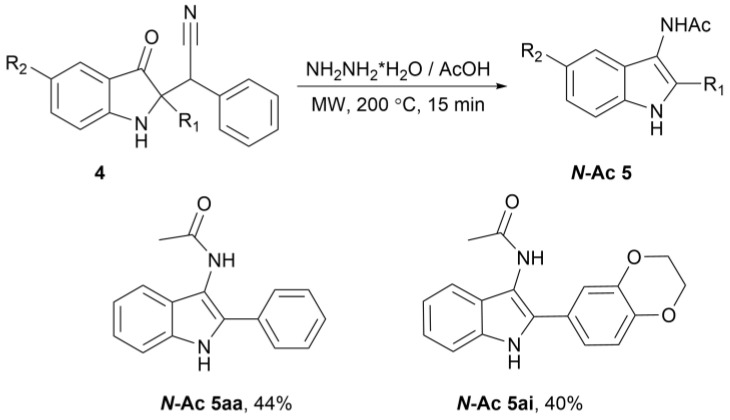
Some examples of *N*-acylated 3-aminoindoles **5**.

Finally, we tested the possibility of subsequent protection of the newly formed amino group by running a reaction in the mixture of hydrazine hydrate and acetic acid. Expectedly, the corresponding *N*-acyl derivatives ***N*-Ac 5aa, 5ai** were received, although in somewhat reduced yields (Scheme 9).

In the end, we would like to share some of our thoughts and observations regarding the shelf stability of the described herein free 3-aminoindoles. It seems that there is a general consensus about the sensitivity of these substances to air and light both in solution and solid and, as a result, the inability to purify them by column chromatography [7,8]. At the same time, we were able to work with most of them (but not sample **19**) rather comfortably, including short and fast (10–15 min) column purifications on silica. The freshly prepared samples, usually light grey or beige, indeed became deep blue with time but even then, their proton NMR spectra showed no signs of significant decomposition. Additionally, while we did not measure the life length of our samples specifically, it might be that the commonly thought tendency of 3-aminoindoles toward oxidative breakdown is somewhat overrated.

## 3. Materials and Methods

### 3.1. General Information

NMR spectra, ^1^H, ^13^C, and ^19^F were measured in solutions of CDCl_3_ or DMSO-*d_6_* on Bruker AVANCE-III HD instrument (at 400, 101, and 376 MHz, respectively). Residual solvent signals were used as internal standards in DMSO*-d_6_* (2.50 ppm for ^1^H and 40.45 ppm for ^13^C nuclei) or CDCl_3_ (7.26 ppm for ^1^H and 77.16 ppm for ^13^C nuclei). HRMS spectra were measured on Bruker maXis impact (electrospray ionization in MeCN solutions, employing HCO_2_Na–HCO_2_H for calibration). IR spectra were measured on FT-IR spectrometer Shimadzu IRAffinity-1S equipped with an ATR sampling module. Spectral data are provided in the Supplementary Materials (S1–S42). Reaction progress, purity of isolated compounds, and R*_f_* values were monitored with TLC on Silufol UV-254 plates. Column chromatography was performed on silica gel (32–63 μm, 60 Å pore size). Melting points were measured with Stuart SMP30 apparatus. All spirocyclic indoles **3** and indolinones **4**, except **4ab** and **4ad**, were synthesized according to the previously reported procedures and were identical to those described [37]. All reagents and solvents were purchased from commercial vendors and used as received.

### 3.2. Preparation of 2-(5-Halide-3-Oxo-2-Phenylindolin-2-Yl)-2-Phenylacetonitrile ***4ab***, ***4ad*** (General Procedure)

These compounds were prepared in analogy to the method described in [37]. A Weaton microreactor equipped with magnetic spin-vane and Mininert valve was charged with a mixture of (E)-(2-Nitrovinyl)benzene (150 mg, 1.0 mmol), corresponding 5-halide-2-phenyl-1H-indole (1.0 mmol), phosphorus acid (1.0 g), and formic acid (1.0 g). The mixture was vigorously stirred for 2 h at room temperature while it turned dark red and homogenized. Then, the mixture was poured into water (50 mL), and the formed crude spirane **3** precipitate was collected and washed with water (4 × 20 mL), dried, and dissolved in ethanol (4 mL). Triethylamine (102 mg, 1.0 mmol) was added, and the resulting solution was stirred at room temperature for 3 h. Crystalline precipitate of crude product was formed, which was collected and purified by preparative column chromatography on silica gel, eluting with ethyl acetate/hexane mixture (1:4).

**2-(5-Fluoro-3-oxo-2-phenylindolin-2-yl)-2-phenylacetonitrile** (**4ab**): yellowish solid, m.p. 200–202 °C, R_f_ 0.56 (EtOAc/hexane, 1:3, *v*/*v*). Yield 274 mg (0.80 mmol, 80%). ^1^H NMR (400 MHz, DMSO-d_6_) δ 8.13 (s, 1H), 7.55 (d, *J* = 7.7 Hz, 2H), 7.47 (t, *J* = 9.3 Hz, 1H), 7.36–7.19 (m, 9H), 7.16 (d, *J* = 9.5 Hz, 1H), 5.31 (s, 1H); ^13^C NMR (101 MHz, DMSO-d_6_) δ 198.7 (d, *J* = 3.7 Hz), 158.8, 155.6 (d, *J* = 237.0 Hz), 134.4, 131.5, 129.4 (2C), 128.5 (2C), 128.5, 128.4 (2C), 126.6, 126.3, 126.3 (2C), 118.9, 117.7 (d, *J* = 7.7 Hz), 114.1 (d, *J* = 7.7 Hz), 109.3 (d, J = 22.7 Hz), 74.3, 43.5; ^19^F NMR (376 MHz, DMSO-d_6_) δ −125.34; FTIR, v_max_: 3351, 2361, 2248, 1698, 1363, 1621, 1558, 1487, 1462, 1330, 1260, 1202 cm^−1^; HRMS (ESI TOF) *m*/*z* calc’d. for C_22_H_15_FN_2_NaO [M+Na]^+^: 365.1061, found: 365.1056 (1.2 ppm).

**2-(5-Chloro-3-oxo-2-phenylindolin-2-yl)-2-phenylacetonitrile** (**4ad**): yellowish solid, m.p. 200–202 °C, R*_f_* 0.49 (EtOAc/hexane, 1:2, *v*/*v*). Yield 215 mg (0.60 mmol, 60%). ^1^H NMR (400 MHz, DMSO-*d*_6_) δ 8.37 (s, 1H), 7.60–7.52 (m, 4H), 7.34–7.18 (m, 8H), 7.15 (d, *J* = 8.8 Hz, 1H), 5.33 (s, 1H); ^13^C NMR (101 MHz, DMSO-*d*_6_) δ 197.9, 160.3, 138.0, 134.3, 131.3, 129.4 (2C), 128.6 (2C), 128.52, 128.50, 128.4 (2C), 126.2 (2C), 123.7, 122.6, 118.8, 118.7, 114.3, 73.9, 43.5; FTIR, *v_max_*: 3298, 2925, 2247, 1693, 1616, 1474, 1301, 1258, 1165, 1056 cm^−1^; HRMS (ESI TOF) *m*/*z* calc’d. for C_22_H_15_ClN_2_NaO^+^ [M+Na]^+^: 381.0765, found: 381.0775 (−2.5 ppm).

### 3.3. Preparation of 2-Aryl-1H-Indol-3-Amine ***5aa–ai*** (General Procedure)

Method A: the corresponding 2-(3-Oxo-2-phenylindolin-2-yl)-2-phenylacetonitrile **4** (1.00 mmol) and 2 mL of hydrazine hydrate were charged in a G10 microwave vial. The vial was sealed and heated in an Anton Paar Monowave 300 microwave apparatus at 200 °C for 15 min. After completion of reaction, vial was opened, and the reaction mixture was concentrated in vacuo. Crude material purified by column chromatography (EtOAc/hexane, 1:4, *v*/*v*).

Method B: the corresponding 2,4′-Diaryl-4′*H*-spiro[indole-3,5′-isoxazole] **3** (1.00 mmol) and 2 mL of hydrazine hydrate were charged in a G10 microwave vial. The vial was sealed and heated in an Anton Paar Monowave 300 microwave apparatus at 200 °C for 15 min. After completion of reaction, vial was opened, and the reaction mixture was concentrated in vacuo. Crude material purified by column chromatography (EtOAc/hexane, 1:4, *v*/*v*).

**2-Phenyl-1*H*-indol-3-amine** (**5aa**): this compound [32] was prepared via both Method A (0.90 mmol, 90%) and Method B (0.80 mmol, 80%). White solid, m.p. 107–108 °C, lit. 110–111 °C, R*_f_* 0.46 (EtOAc/hexane, 1:4, *v*/*v*). ^1^H NMR (400 MHz, DMSO-*d_6_*) δ 10.52 (s, 1H), 7.85–7.77 (m, 2H), 7.69 (d, *J* = 7.9 Hz, 1H), 7.44 (t, *J* = 7.8 Hz, 2H), 7.27 (d, *J* = 8.1 Hz, 1H), 7.20 (t, *J* = 7.4 Hz, 1H), 7.11–7.02 (m, 1H), 6.96–6.87 (m, 1H), 4.50 (s, 2H); ^13^C NMR (CPD) (101 MHz, DMSO-*d_6_*) δ 135.0, 133.6, 128.7 (2C), 125.1 (2C), 125.0, 123.0 (2C), 122.0, 118.9, 118.3, 117.4, 110.9; ^13^C NMR (DEPT135) (101 MHz, DMSO-*d_6_*) δ 128.5 (2C), 124.83 (2C), 124.78, 121.7, 118.1, 117.1, 110.6; ^13^C NMR (DEPTQ) (101 MHz, DMSO-*d_6_*) δ 135.0, 133.6, 128.7 (2C), 125.1 (2C), 125.0, 123.0 (2C), 122.0, 118.9, 118.3, 117.4, 110.9; FTIR, *v_max_*: 3646, 3503, 3351, 3198, 1887, 1769, 1684, 1600, 1489, 1457, 1378, 1245 cm^−1^; HRMS (ESI TOF) *m*/*z*: calc’d for C_14_H_13_N_2_ [M+H]^+^: 209.1073, found 209.1069 (2.1 ppm).

**5-Fluoro-2-phenyl-1*H*-indol-3-amine** (**5ab**): this compound was prepared via Method A (0.77 mmol, 77%). White solid, m.p. 105–106 °C, R*_f_* 0.33 (EtOAc/hexane, 1:3, *v*/*v*). ^1^H NMR (400 MHz, DMSO-*d_6_*) δ 10.58 (br. s, 1H), 7.86–7.72 (m, 2H), 7.51–7.40 (m, 3H), 7.25–7.17 (m, 2H), 6.87 (td, *J* = 9.2, 2.6 Hz, 1H), 4.47 (br. s, 2H); ^13^C{^1^H} NMR (101 MHz, DMSO-*d_6_*) δ 157.2, 154.9, 132.4 (d, *J* = 167.3 Hz), 128.7 (2C), 125.5, 125.2 (2C), 123.1 (d, *J* = 4.8 Hz), 122.8 (d, *J* = 9.9 Hz), 121.2, 111.7 (d, *J* = 9.5 Hz), 109.9 (d, *J* = 26.0 Hz), 103.0 (d, *J* = 23.8 Hz); ^19^F NMR (376 MHz, DMSO-*d_6_*) δ −126.28 (s); FTIR, *v_max_*: 3629, 3371, 2993, 1922, 1771, 1688, 1522, 1248, 1174, 1061 cm^−1^; HRMS (ESI TOF) *m*/*z*: calc’d for C_14_H_11_FN_2_ [M+H]^+^: 227.0979, found 227.0976 (1.2 ppm).

**5-Isopropyl-2-phenyl-1*H*-indol-3-amine** (**5ac**): this compound was prepared via Method A (0.82 mmol, 82%). White solid, m.p. 77–79 °C, R*_f_* 0.56 (EtOAc/hexane, 1:3, *v*/*v*). ^1^H NMR (400 MHz, DMSO-*d_6_*) δ 10.34 (br. s, 1H), 7.79 (dd, *J* = 8.5, 1.3 Hz, 2H), 7.53 (s, 1H), 7.45–7.39 (m, 2H), 7.20–7.13 (m, 2H), 6.96 (dd, *J* = 8.4, 1.8 Hz, 1H), 4.42 (s, 2H), 3.02–2.83 (m, 1H), 1.28 (s, 3H), 1.26 (s, 3H); ^13^C{^1^H} NMR (101 MHz, DMSO-*d_6_*) δ 137.5, 133.8, 133.7, 128.7 (2C), 125.0 (2C), 124.9, 123.0, 122.9, 121.3, 119.2, 115.00, 110.6, 33.7, 24.3 (2C); FTIR, *v_max_*: 3381, 2950, 1922, 1827, 1655, 1524, 1459, 1240 cm^−1^; HRMS (ESI TOF) *m*/*z*: calc’d for C_17_H_19_N_2_ [M+H]^+^: 251.1543, found 251.1543 (1.2 ppm).

**5-Chloro-2-phenyl-1*H*-indol-3-amine** (**5ad**): this compound [2] was prepared via Method A (0.85 mmol, 85%). Yellow solid, m.p. 124–125 °C, lit. 125–127 °C, R*_f_* 0.65 (EtOAc/hexane, 1:2, *v*/*v*). ^1^H NMR (400 MHz, DMSO-*d_6_*) δ 10.72 (s, 1H), 7.79 (d, *J* = 8.3 Hz, 3H), 7.45 (t, *J* = 7.6 Hz, 2H), 7.29–7.18 (m, 2H), 7.03 (dd, *J* = 8.6, 2.1 Hz, 1H), 4.58 (s, 2H); ^13^C NMR (101 MHz, DMSO-*d*_6_) δ 133.2, 133.1, 128.7 (2C), 125.5, 125.2 (2C), 123.8, 122.6, 121.9, 121.6, 120.5, 117.7, 112.3; FTIR, *v_max_*: 3742, 3627, 3324, 3066, 2358, 1686, 1558, 1507, 1455, 1352 cm^−1^; HRMS (ESI TOF) *m*/*z* calc’d. for C_14_H_12_ClN_2_ [M+H]^+^: 243.0679, found: 243.0684 (1.8 ppm).

**2-(Naphthalen-2-yl)-1*H*-indol-3-amine** (**5ae**): this compound was prepared via Method A (0.70 mmol, 70%). Yellow solid, m.p. 183–186 °C, R*_f_* 0.53 (EtOAc/hexane, 1:2, *v*/*v*). ^1^H NMR (400 MHz, DMSO-*d*_6_) δ 10.64 (s, 1H), 8.24 (d, *J* = 1.8 Hz, 1H), 8.04 (dd, *J* = 8.7, 1.8 Hz, 1H), 7.96 (d, *J* = 8.7 Hz, 1H), 7.93–7.85 (m, 2H), 7.71 (d, *J* = 7.9 Hz, 1H), 7.51 (ddd, *J* = 8.2, 6.8, 1.4 Hz, 1H), 7.45 (ddd, *J* = 8.0, 6.8, 1.3 Hz, 1H), 7.27 (d, *J* = 8.1 Hz, 1H), 7.08 (ddd, *J* = 8.1, 6.9, 1.2 Hz, 1H), 6.92 (ddd, *J* = 7.9, 6.9, 1.0 Hz, 1H), 4.69 (s, 2H); ^13^C NMR (101 MHz, DMSO-*d*_6_) δ 135.3, 133.6, 131.2, 130.9, 128.0, 127.7, 127.6, 126.4, 125.2, 124.2, 123.8, 122.9, 122.5, 122.2, 118.7, 118.4, 117.4, 110.9; FTIR, *v_max_*: 3360, 3157, 3062, 2922, 2851, 1908, 1871, 1608, 1548, 1488 cm^−1^; HRMS (ESI TOF) *m*/*z* calc’d. for C_18_H_15_N_2_ [M+H]^+^: 259.1223, found: 259.1230 (2.8 ppm).

**2-(4-Methoxyphenyl)-1*H*-indol-3-amine** (**5af**): this compound [7] was prepared via Method A (0.68 mmol, 68%). White solid, m.p. 113–115 °C, lit. 108–110 °C, R*_f_* 0.25 (EtOAc/hexane, 1:3, *v*/*v*). ^1^H NMR (400 MHz, DMSO-*d_6_*) δ 10.44 (br. s, 1H), 7.74 (d, *J* = 8.8 Hz, 2H), 7.62 (d, *J* = 7.8 Hz, 1H), 7.22 (d, *J* = 8.1 Hz, 1H), 7.06–6.99 (m, 3H), 6.89 (t, *J* = 7.4 Hz, 1H), 4.30 (br. s, 2H), 3.79 (s, 3H); ^13^C{^1^H} NMR (101 MHz, DMSO-*d_6_*) δ 157.1, 134.6, 126.6 (2C), 126.3, 123.3, 121.4, 121.3, 119.5, 117.9, 117.4, 114.2 (2C), 110.7, 55.2; FTIR, *v_max_*: 3619, 3265, 2957, 1769, 1749, 1562, 1456, 1374, 1237, 1085 cm^−1^; HRMS (ESI TOF) *m*/*z*: calc’d for C_15_H_15_N_2_O [M+H]^+^: 239.1179, found 239.1184 (−2.3 ppm).

**2-(*p*-Tolyl)-1*H*-indol-3-amine** (**5ag**): this compound [7] was prepared via Method A (0.65 mmol, 65%). White solid, m.p. 149–151 °C, lit. 149–151 °C, R*_f_* 0.61 (EtOAc/hexane, 1:2, *v*/*v*). ^1^H NMR (400 MHz, DMSO-*d*_6_) δ 10.45 (s, 1H), 7.68 (d, *J* = 7.9 Hz, 2H), 7.63 (d, *J* = 7.9 Hz, 1H), 7.23 (dd, *J* = 11.5, 7.8 Hz, 3H), 7.02 (ddd, *J* = 8.1, 6.9, 1.2 Hz, 1H), 6.88 (ddt, *J* = 8.1, 7.0, 1.1 Hz, 1H), 4.39 (s, 2H), 2.33 (s, 3H); ^13^C NMR (101 MHz, DMSO-*d*_6_) δ 134.8, 134.2, 130.8, 129.3 (2C), 125.0 (2C), 123.0, 122.3, 121.7, 119.1, 118.1, 117.3, 110.7, 20.8; FTIR, *v_max_*: 3742, 3627, 3324, 3066, 2358, 1686, 1558, 1507, 1455, 1352 cm^−1^; HRMS (ESI TOF) *m*/*z* calc’d. for C_15_H_15_N_2_ [M+H]^+^: 223.1225, found: 223.1230 (2.1 ppm).

**2-(5,6,7,8-Tetrahydronaphthalen-2-yl)-1*H*-indol-3-amine** (**5ah**): this compound was prepared via Method A (0.9 mmol, 90%). Yellowish solid, m.p. 164–167 °C, R*_f_* 0.63 (EtOAc/hexane, 1:2, *v*/*v*). ^1^H NMR (400 MHz, CDCl_3_) δ 7.64 (s, 1H), 7.54 (d, *J* = 7.8 Hz, 1H), 7.41 (dd, *J* = 7.9, 2.0 Hz, 1H), 7.36 (d, *J* = 1.8 Hz, 1H), 7.33 (d, *J* = 8.0 Hz, 1H), 7.21 (t, *J* = 7.0 Hz, 2H), 7.13 (t, *J* = 7.4 Hz, 1H), 3.52 (s, 2H), 2.86 (dt, *J* = 11.0, 5.1 Hz, 4H), 2.09–1.57 (m, *J* = 4.0 Hz, 4H); ^13^C NMR (101 MHz, CDCl_3_) δ 138.2, 135.9, 134.9, 130.2, 130.1, 126.7, 123.5, 123.4, 122.6, 121.4, 120.7, 119.1, 117.3, 111.0, 29.7, 29.3, 23.32, 23.29; FTIR, *v_max_*: 3332, 2938, 2370, 1578, 1472, 1360, 1314, 1254, 1206, 1023 cm^−1^; HRMS (ESI TOF) *m*/*z* Calc’d. for C_18_H_19_N_2_ [M+H]^+^: 263.1544, found: 2639.1543 (−0.4 ppm).

**2-(2,3-Dihydrobenzo[b][1,4]dioxin-6-yl)-1*H*-indol-3-amine** (**5ai**): this compound was prepared via Method A (0.83 mmol, 83%). Pale yellow solid, m.p. 101–102 °C, R*_f_* 0.15 (EtOAc/hexane, 1:3, *v*/*v*). ^1^H NMR (400 MHz, DMSO-*d_6_*) δ 10.41 (br. s, 1H), 7.61 (d, *J* = 7.9 Hz, 1H), 7.34–7.26 (m, 2H), 7.23–7.16 (m, 1H), 7.04–6.98 (m, 1H), 6.95–6.84 (m, 2H), 4.28 (br. s, 2H), 4.28–4.19 (m, 4H); ^13^C{^1^H} NMR (101 MHz, DMSO-*d_6_*) δ 143.6, 141.3, 134.6, 127.1, 123.2, 121.7, 121.5, 119.1, 118.5, 118.0, 117.4, 117.3, 113.8, 110.7, 64.3, 64.2; FTIR, *v_max_*: 3619, 3265, 2957, 1769, 1749, 1562, 1456, 1374, 1237, 1085 cm^−1^; HRMS (ESI TOF) *m*/*z*: calc’d for C_16_H_15_N_2_O_2_ [M+H]^+^: 267.1128, found 267.1121 (2.8 ppm).

**1-Methyl-2-phenyl-1*H*-indol-3-amine** (**19**): this compound was prepared via Method A employing the corresponding *N*-methylated indolinone **18** (338 mg, 1.00 mmol) in a yield of 91 mg (0.41 mmol, 41%). Purification was performed by column chromatography (EtOAc/hexane, 1:4, *v*/*v*). The titled compound was obtained as white solid, R*_f_* 0.36 (EtOAc/hexane, 1:4, *v*/*v*). ^1^H NMR (400 MHz, CDCl_3_) δ 7.61–7.49 (m, 3H), 7.49–7.42 (m, 2H), 7.41–7.37 (m, 1H), 7.33–7.29 (m, 1H), 7.27–7.24 (m, 1H), 7.14–7.09 (m, 1H), 3.61 (s, 3H), 2.89 (s, 2H); ^13^C{^1^H} NMR (101 MHz, CDCl_3_) δ 136.4, 131.5, 130.0, 129.0 (2C), 127.5, 125.2, 122.3, 121.8, 120.7, 118.7, 117.6, 109.5 (2C), 31.2; FTIR, *v_max_*: 3459, 3356, 3321, 3066, 1651, 1531, 1511, 1431, 1311, 1167 cm^−1^; HRMS (ESI TOF) *m*/*z*: calc’d for C_15_H_15_N_2_Na [M+Na]^+^: 223.1230, found 223.1228 (1.0 ppm).

**1-methyl-2-phenyl-1*H*-indole** (**J**): the corresponding *N*-methylated indolinone **18** (0.30 mmol), hydroxylamine hydrochloride (0.3 mmol), triethylamine (0.3 mmol), and 2 mL xylene were charged in a G10 microwave vial. The vial was sealed and heated in an Anton Paar Monowave 300 microwave apparatus at 200 °C for 30 min. After completion of reaction vial was opened and the reaction mixture was concentrated in vacuo. Crude material purified by column chromatography (hexane) in a yield of 9 mg (0.045 mmol, 15%). The titled compound was obtained as light-yellow solid, m.p. 99–101 °C, R*_f_* 0.71 (EtOAc/hexane, 1:10, *v*/*v*). ^1^H NMR (400 MHz, CDCl_3_) δ 7.71 (d, *J* = 7.9 Hz, 1H), 7.58 (d, J = 7.6 Hz, 2H), 7.53 (t, *J* = 7.5 Hz, 2H), 7.45 (dd, *J* = 16.2, 7.6 Hz, 2H), 7.32 (t, *J* = 7.6 Hz, 1H), 7.22 (t, *J* = 7.4 Hz, 1H), 6.64 (s, 1H), 3.80 (s, 3H); ^13^C NMR (101 MHz, CDCl_3_) δ 141.7, 138.4, 132.9, 129.5 (2C), 128.6 (2C), 128.1, 128.0, 121.8, 120.6, 120.0, 109.7, 101.7, 31.3.

### 3.4. Preparation of N-(2-Aryl-1H-Indol-3-yl)acetamide N-Ac ***5aa*** and N-Ac ***5ai*** (General Procedure)

The corresponding 2-(3-Oxo-2-arylindolin-2-yl)-2-phenylacetonitrile **4** (1.00 mmol), 1 mL of glacial acetic acid, and 2 mL of hydrazine hydrate were charged in a G10 microwave vial. The vial was sealed and heated in an Anton Paar Monowave 300 microwave apparatus at 200 °C for 15 min. After completion of reaction, vial was opened, and the reaction mixture was concentrated in vacuo. Crude material purified by column chromatography (EtOAc/hexane, 2:1, *v*/*v*).

***N*-(2-Phenyl-1*H*-indol-3-yl)acetamide** (***N*-Ac 5aa**): white solid, R*_f_* 0.17 (EtOAc/hexane, 1:1, *v*/*v*). ^1^H NMR (400 MHz, DMSO-*d_6_*) δ 11.38 (s, 1H), 9.44 (s, 1H), 7.77 (d, *J* = 8.2 Hz, 2H), 7.48 (t*, J* = 7.7 Hz, 2H), 7.45–7.29 (m, 3H), 7.12 (t, *J* = 7.6 Hz, 1H), 7.00 (t, *J* = 7.5 Hz, 1H), 2.09 (s, 3H); ^13^C{^1^H} NMR (101 MHz, DMSO-*d_6_*) δ 169.7, 134.5, 131.7, 131.3, 128.8 (2C), 127.5, 126.7 (2C), 126.2, 122.0, 119.1, 118.5, 111.4, 110.8, 22.8; FTIR, *v_max_*: 3435, 3265, 2918, 2863, 1648, 1458, 1377, 1338, 1242, 1191, 1148, 1113, 1075 cm^−1^; HRMS (ESI TOF) *m*/*z*: calc’d for C_16_H_14_N_2_ONa [M+Na]^+^: 273.0998, found 273.0995 (1.2 ppm).

***N*-(2-(2,3-Dihydrobenzo[b]**[1,4]**dioxin-6-yl)-1*H*-indol-3-yl)acetamide** (***N*-Ac 5ai**): white solid, R*_f_* 0.29 (EtOAc/hexane, 2:1, *v*/*v*). ^1^H NMR (400 MHz, DMSO-*d_6_*) δ 11.25 (s, 1H), 9.37 (s, 1H), 7.33 (d, *J* = 8.1 Hz, 1H), 7.30–7.23 (m, 3H), 7.09 (t, *J* = 6.9 Hz, 1H), 6.96 (d, *J* = 8.3 Hz, 2H), 4.28 (s, 4H), 2.08 (s, 3H); ^13^C{^1^H} NMR (101 MHz, DMSO-*d_6_*) δ 169.7, 143.5, 143.1, 134.3, 131.1, 126.3, 125.0, 121.7, 119.9, 119.0, 118.2, 117.4, 115.3, 111.2, 110.0, 64.3, 64.2, 22.9; FTIR, *v_max_*: 3259, 1655, 1585, 1511, 1494, 1459, 1372, 1340, 1282, 1246, 1171, 1126, 1063 cm^−1^; HRMS (ESI TOF) *m*/*z*: calc’d for C_18_H_16_N_2_O_3_Na [M+Na]^+^: 331.1053, found 331.1046 (2.2 ppm).

## 4. Conclusions

A novel preparative method for the synthesis of diverse 3-aminoindoles **5** based on a microwave-assisted cascade reaction of 2-(3-oxoindolin-2-yl)-2-arylacetonitriles **4** with hydrazine hydrate was developed. Alternatively, the same transformation could also be carried out from 4′-phenyl-4′H-spiro[indole-3,5′-isoxazoles] **3**. Considering that such spirocyclic indoles **3** could be obtained in a single step from commonly available indoles **1** and nitrostyrene **2**, the overall sequence provides a very convenient and affordable route to generally not easily available 3-aminoindoles.

## Figures and Tables

**Table 1:** Reaction of **4aa** with amino binucleophiles RXHNH_2_ under different conditions.

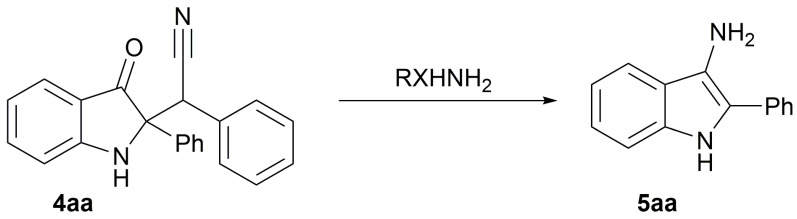			
#	Binucleophile, RXHNH_2_	Conditions	Yield 5aa(Isolated), %
1	Ethylenediamine	1-butanol, MW 200 °C, 0.5 h	37
2	Propane-1,2-diamine	1-butanol, MW 200 °C, 0.5 h	41
3	Formamidine acetate	1-butanol, MW 185 °C, 1 h	decomposed
4	Thiosemicarbazide	Xylene, MW 180 °C, 0.5 h	15
5	2-hydrazinylpyridine	Xylene, MW 160 °C, 0.5 h	20
6	Phenylhydrazine	Xylene, MW 200 °C, 0.5 h	35
7	Tosylhydrazine	Xylene, MW 200 °C, 0.5 h	39
8	Benzohydrazide	Xylene, MW 200 °C, 0.5 h	26
9	Hydrazine hydrate	neat, MW 200 °C, 15 min	90
10	Hydroxylamine	Xylene, MW 200 °C, 0.5 h	n.d.

## Data Availability

The supporting information includes NMR spectral and HRMS charts.

## Supplementary Materials

The following supporting information that includes 1H, 13C, and 19F NMR spectral and HRMS charts can be downloaded at: https://www.mdpi.com/article/10.3390/molecules28093657/s1.

## Sample Availability

Not available.
