# Supplementary material for: A Two-Step Synthesis of Unprotected 3-Aminoindoles via Post Functionalization with Nitrostyrene
Source: Molecules. 2023 Apr 23;28(9):3657. doi: 10.3390/molecules28093657 (PMC10180116; doi:10.3390/molecules28093657)
Supplement: Supplementary file 1 [file molecules-28-03657-s001.zip › molecules-2347001-supplementary.pdf]

Electronic Supplementary Material (ESI)

A two-step synthesis of unprotected 3-aminoindoles via post functionalization with nitrostyrene

Nicolai A. Aksenov, Nikolai A. Arutiunov, Igor A. Kurenkov, Vladimir V. Malyuga, Dmitrii A. Aksenov, Daria S. Momotova, Anna M. Zatsepilina, Elizaveta A. Chukanova, Alexander V. Leontiev, and Alexander V. Aksenov

Department of Chemistry, North Caucasus Federal University, 1a Pushkin St., Stavropol 355009, Russian Federation

Supporting Information

NMR Spectral Charts……………………………………………………………………………………………S2
^{1}H and ^{13}C NMR spectral charts for indolinones **4ab**, **4ad**…...……………………………………………………….......S2
^{1}H and ^{13}C NMR spectral charts for 3-aminoindole **5aa-ai**, **19**, ***N*-Ac 5aa** and ***N*-Ac 5ai**…………………………….……...S7
^{1}H and ^{13}C NMR spectral charts for 1-methyl-2-phenyl-1*H*-indole **J.**…………………………………………...……...S34
HRMS spectral chart for **4ab**, **4ad, 5aa-ai**, **19**, ***N*-Ac 5aa** and ***N*-Ac 5ai**.............……………………………………………S36

^{1}H and ^{13}C NMR spectral charts for 2-(5-halide-3-oxo-2-phenylindolin-2-yl)-2-phenylacetonitrile **4ab**, **4ad**

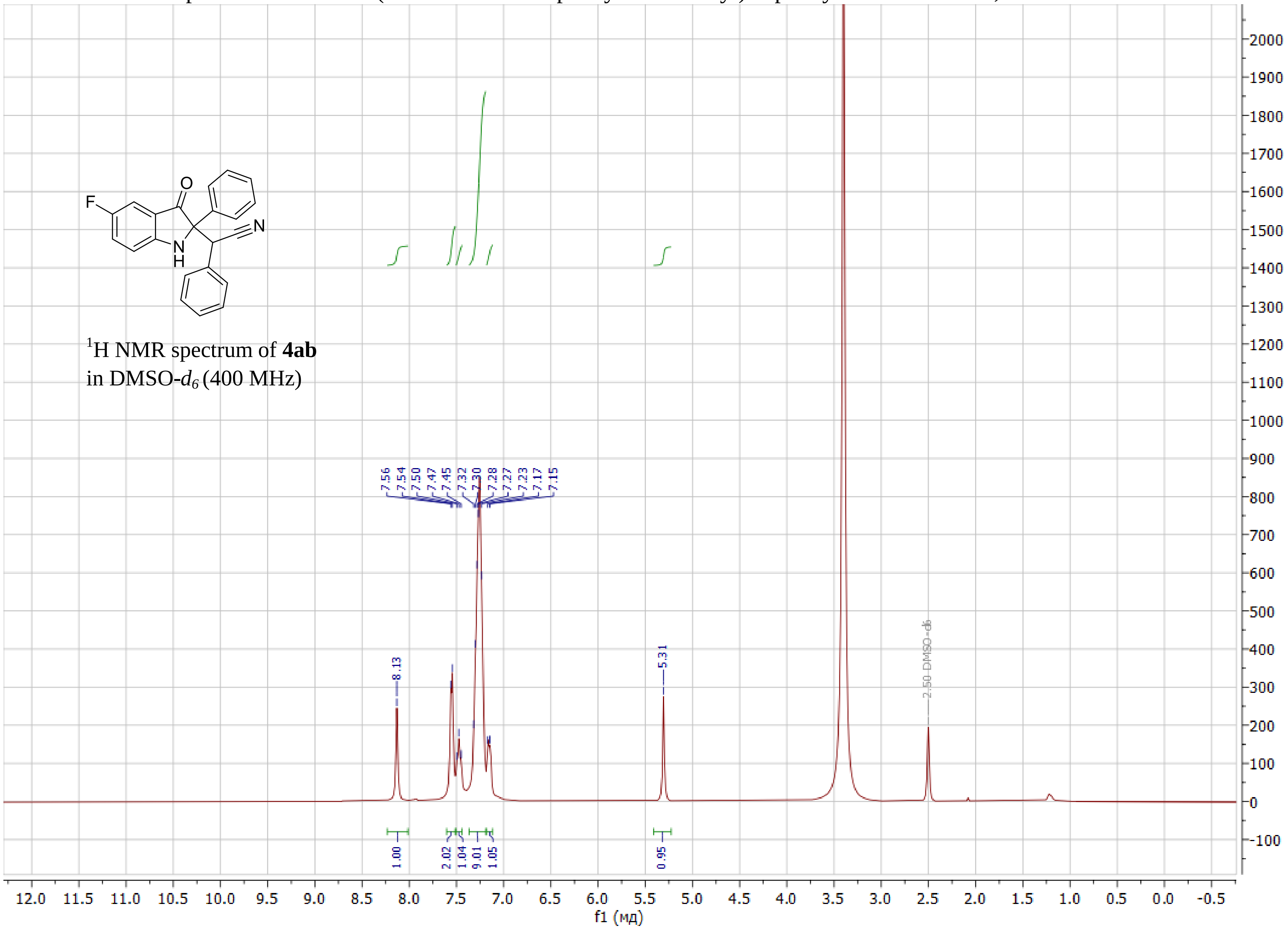

^{1}H NMR spectrum of **4ab** in DMSO-d_6 (400 MHz)

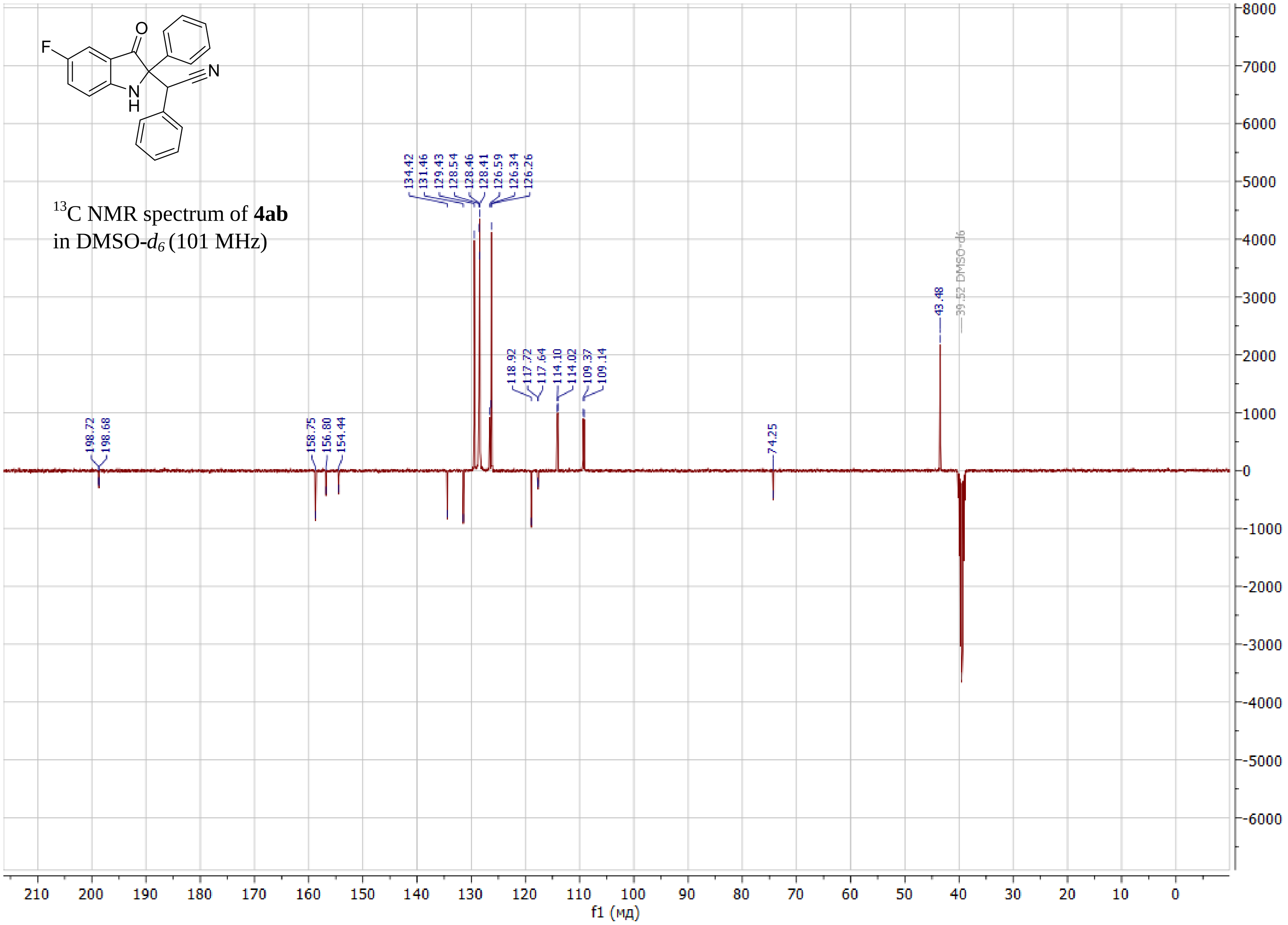

^{13}C NMR spectrum of **4ab** in DMSO-d_6 (101 MHz)

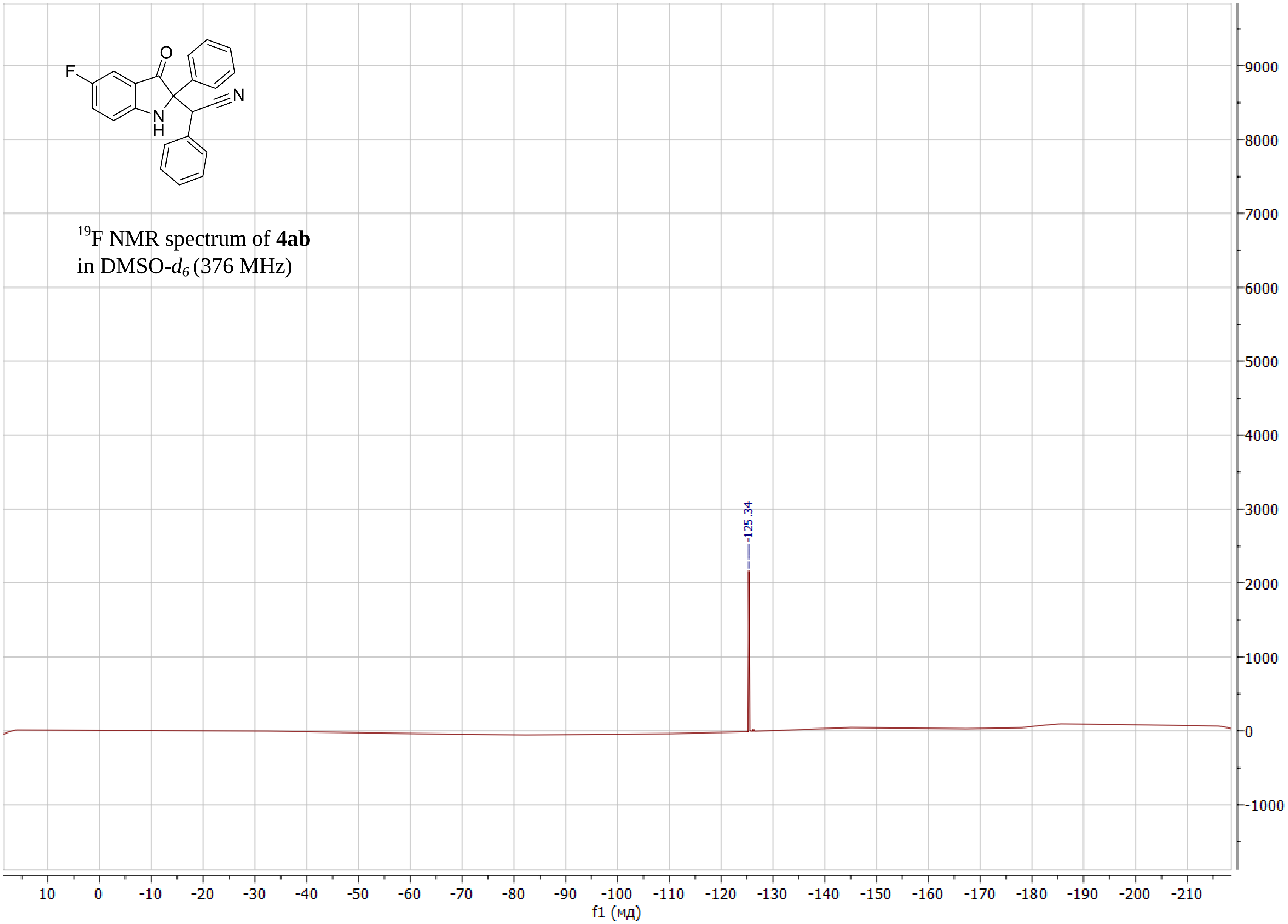

^{19}F NMR spectrum of **4ab** in DMSO-d_6 (376 MHz)

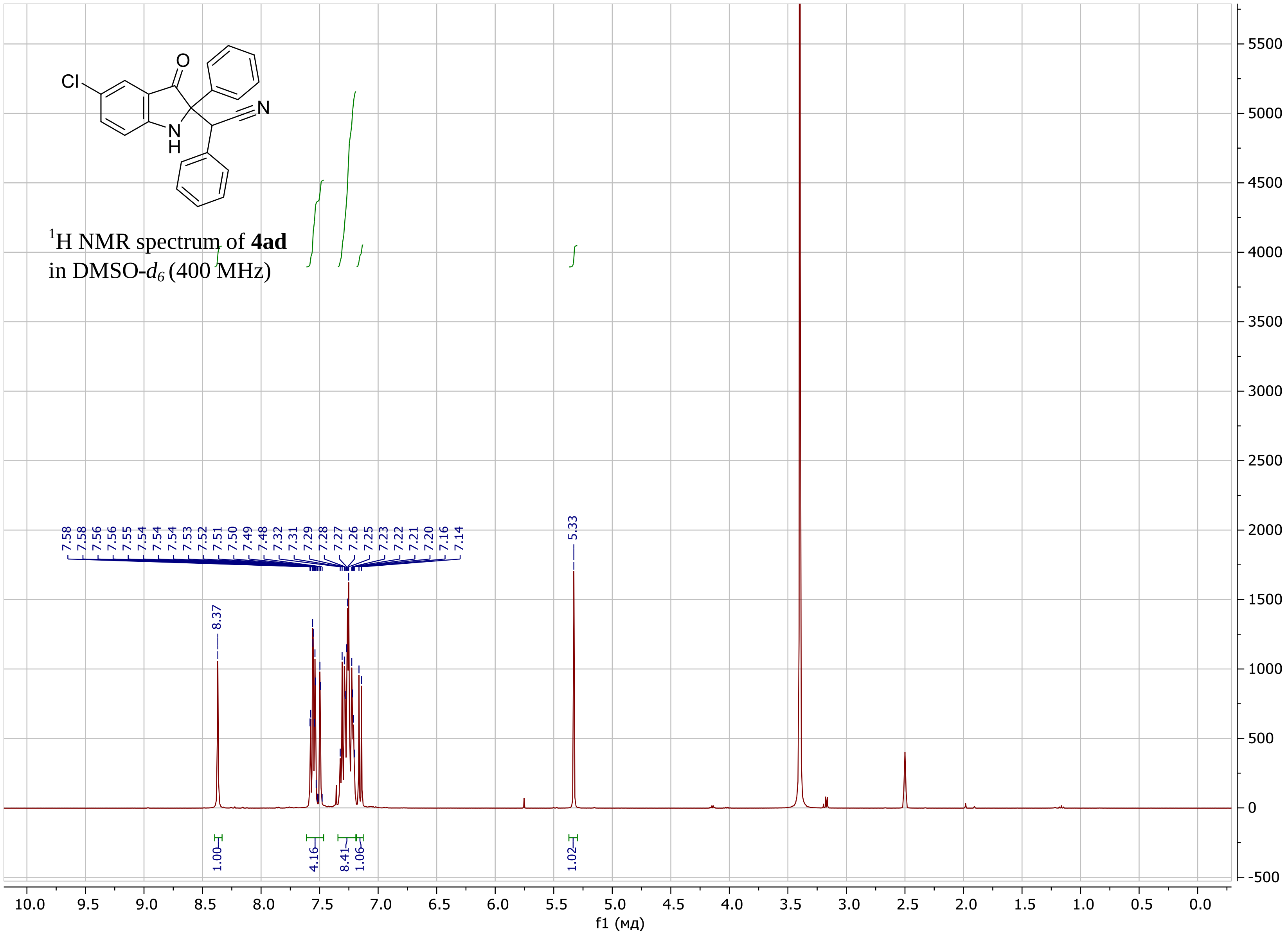

^{1}H NMR spectrum of **4ad** in DMSO-d_6 (400 MHz)

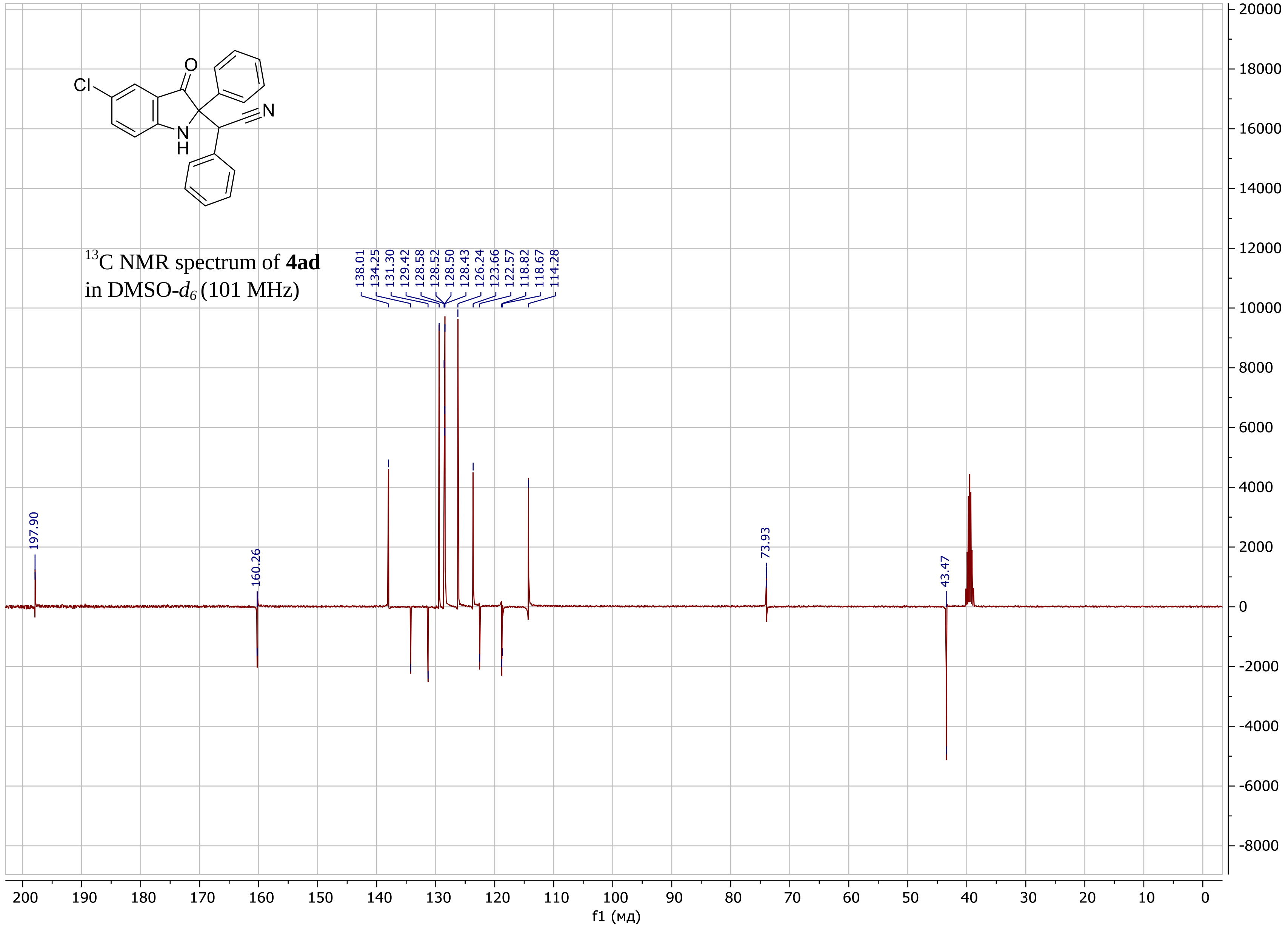

13C NMR spectrum of 4ad
in DMSO-d6 (101 MHz)
138.01
134.25
131.30
129.42
128.58
128.52
128.50
128.43
126.24
123.66
122.57
118.82
118.67
114.28
197.90
160.26
73.93
43.47
f1 (мд)

^{1}H and ^{13}C NMR spectral charts for 2-aryl-1H-indol-3-amines **5aa-5ai**, **19**, ***N*-Ac 5aa** and ***N*-Ac 5ai**

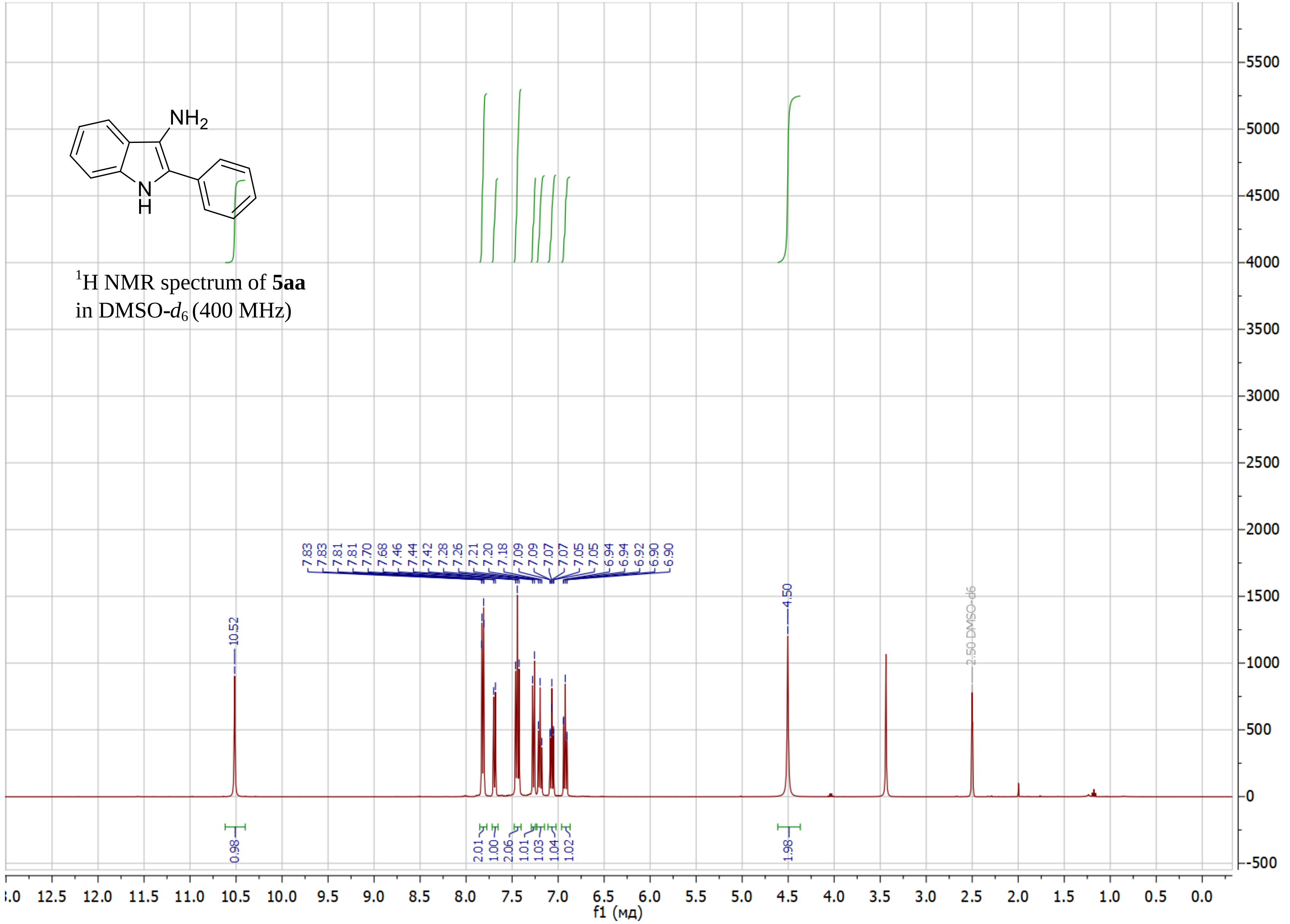

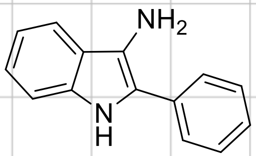

^{13}C NMR (CPD) spectrum of **5aa**
in DMSO-d_6 (101 MHz)

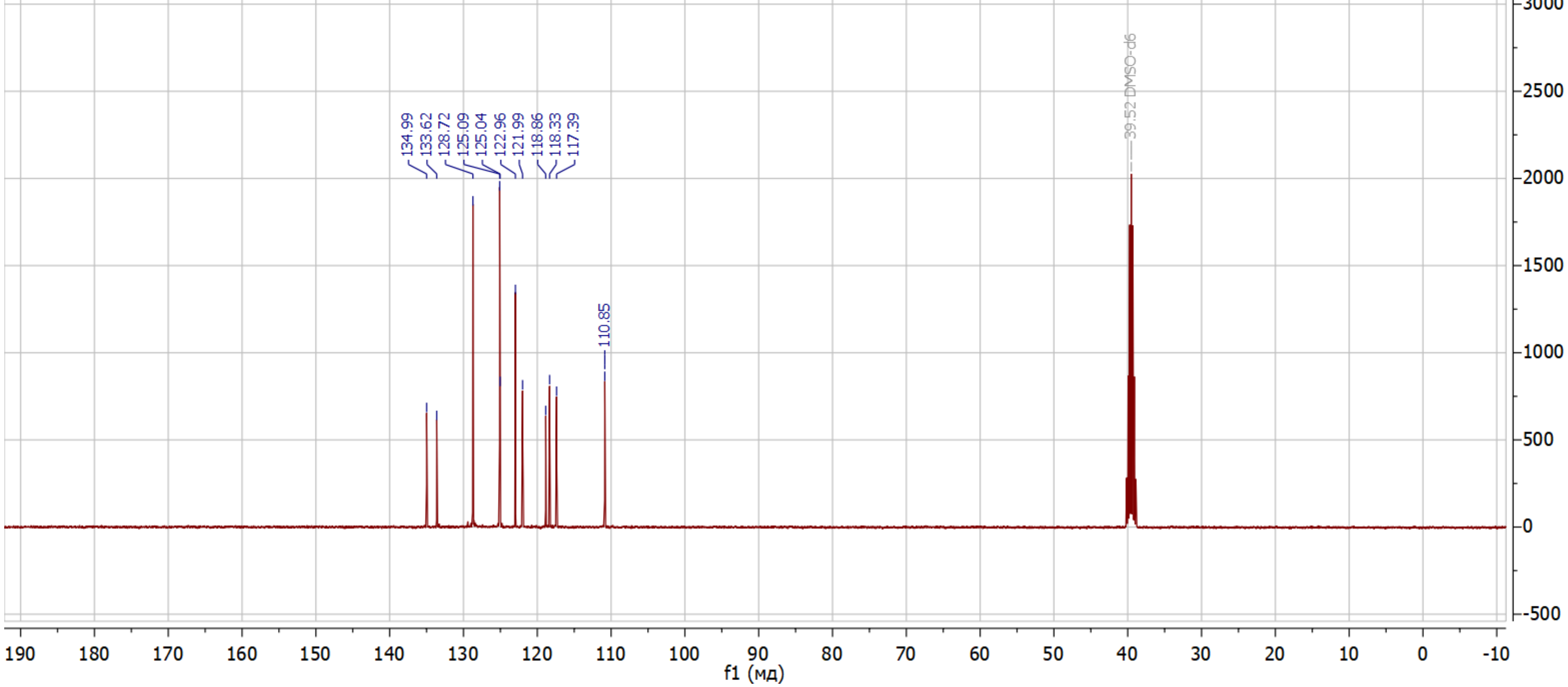

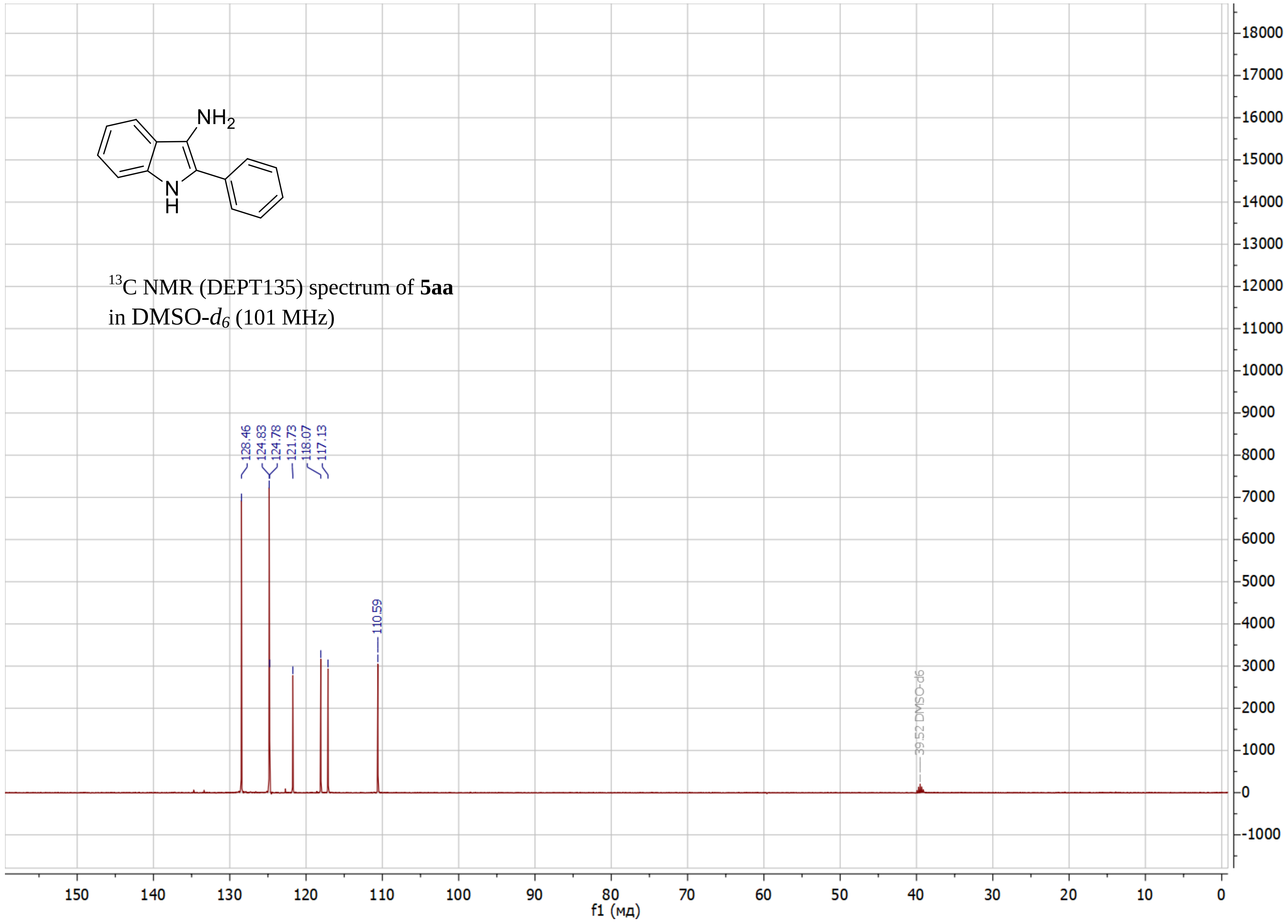
NH2
N
H
13C NMR (DEPT135) spectrum of 5aa
in DMSO-d6 (101 MHz)
128.46
124.83
124.78
121.73
118.07
117.13
110.59
39.52 DMSO-d6
f1 (мд)

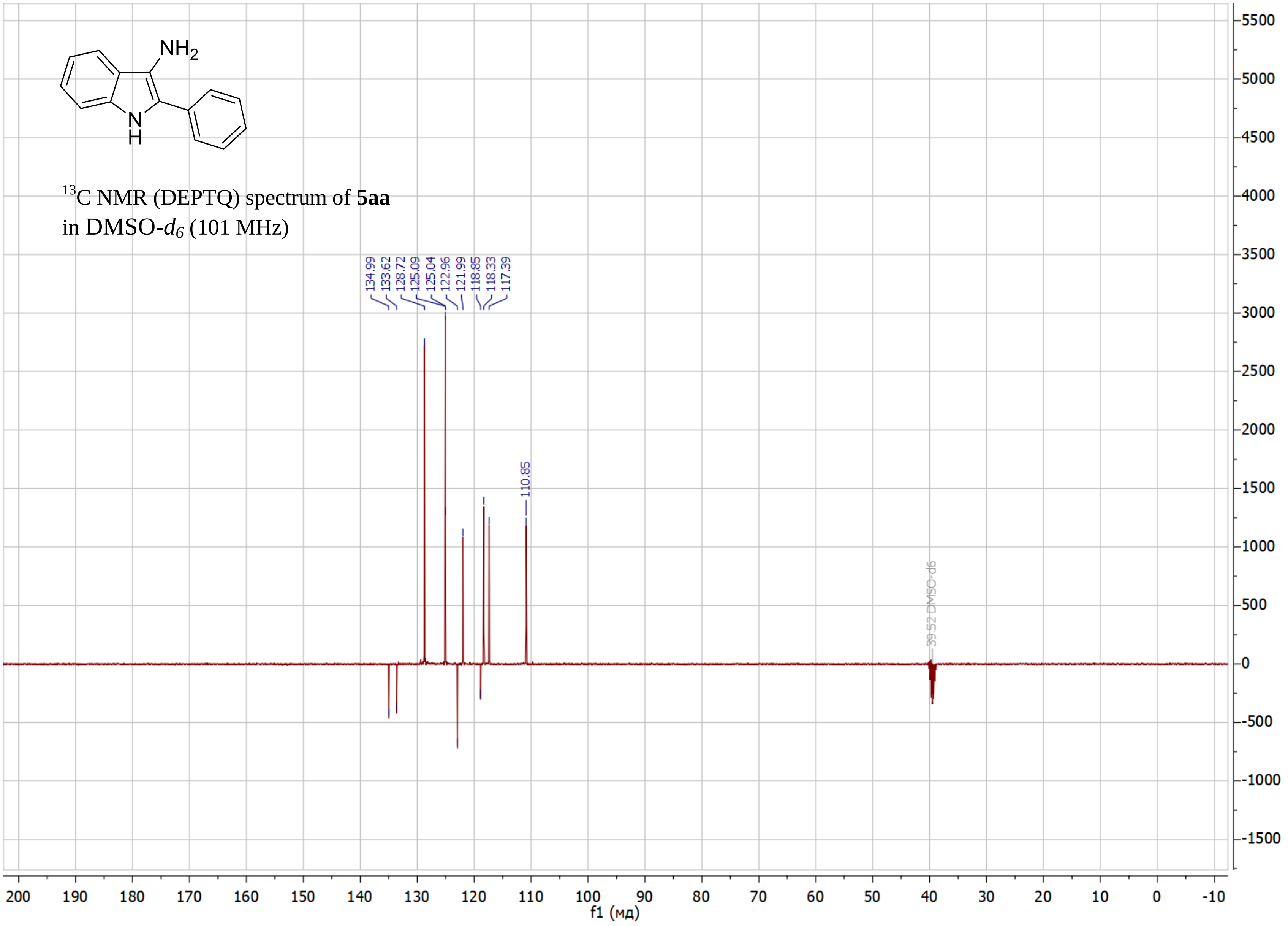
NH2
N
H
13C NMR (DEPTQ) spectrum of 5aa
in DMSO-d6 (101 MHz)
134.99
133.62
128.72
125.09
125.04
122.96
121.99
118.85
118.33
117.39
110.85
39.52 DMSO-d6
f1 (мд)

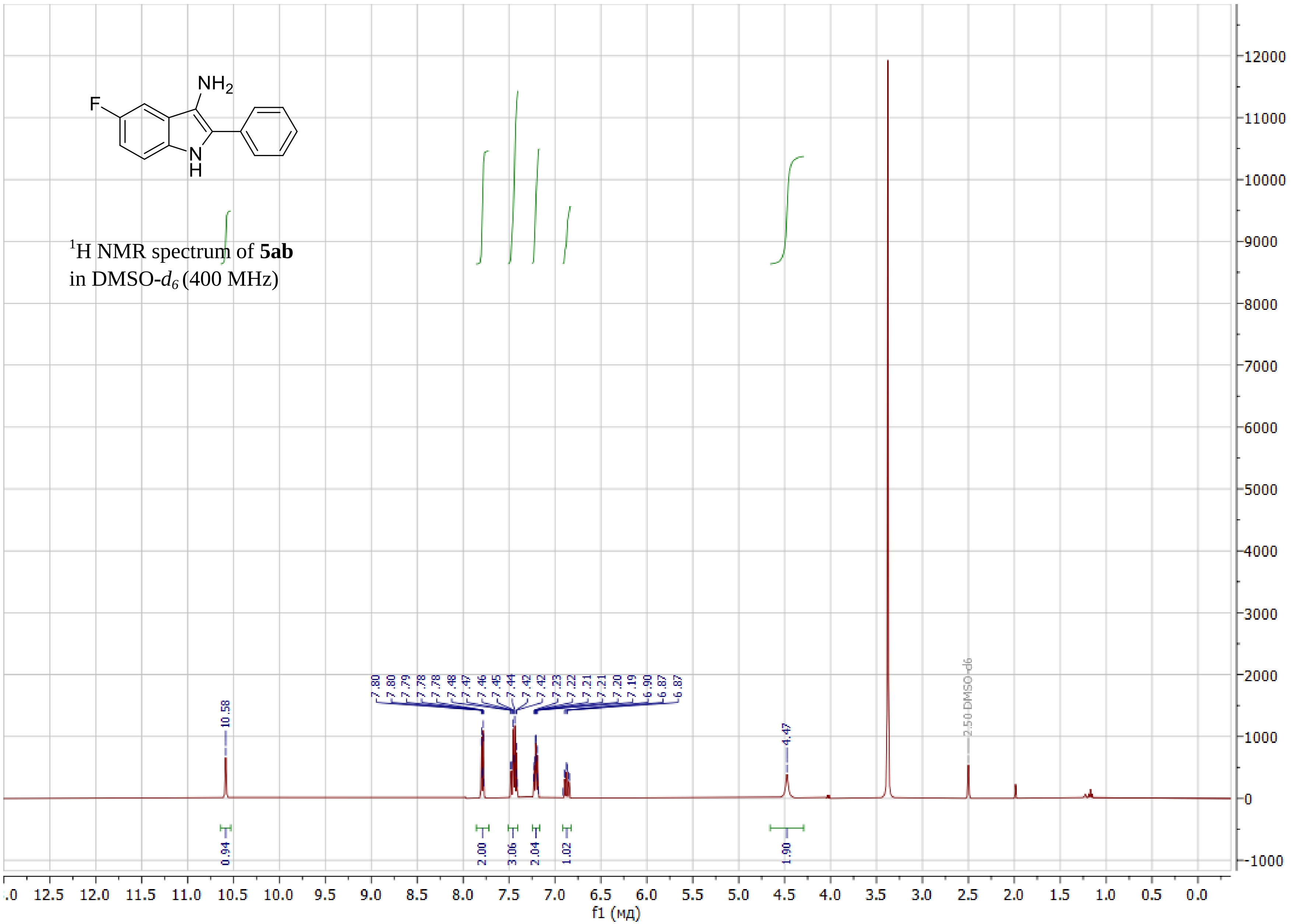

NH2
F
N
H
^{1}H NMR spectrum of **5ab**
in DMSO-d_6 (400 MHz)
10.58
7.80
7.80
7.79
7.78
7.78
7.48
7.47
7.46
7.45
7.44
7.42
7.42
7.23
7.22
7.21
7.21
7.20
7.19
6.90
6.87
6.87
4.47
2.50 DMSO-d6
0.94
2.00
3.06
2.04
1.02
1.90
f1 (мд)

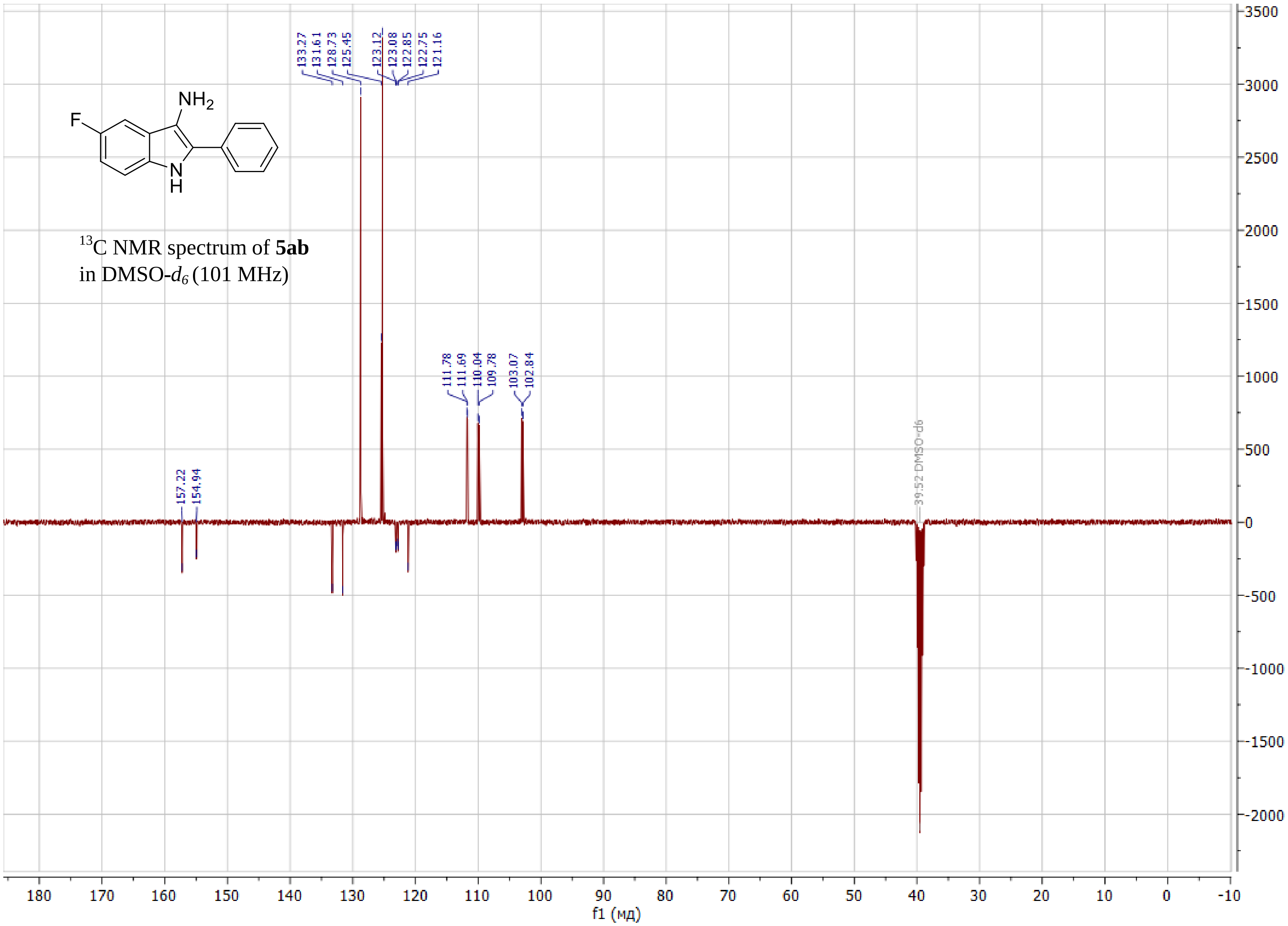

NH2
F
N
H
13C NMR spectrum of 5ab
in DMSO-d6 (101 MHz)
133.27
131.61
128.73
125.45
123.12
123.08
122.85
122.75
121.16
111.78
111.69
110.04
109.78
103.07
102.84
157.22
154.94
39.52 DMSO-d6
f1 (мд)

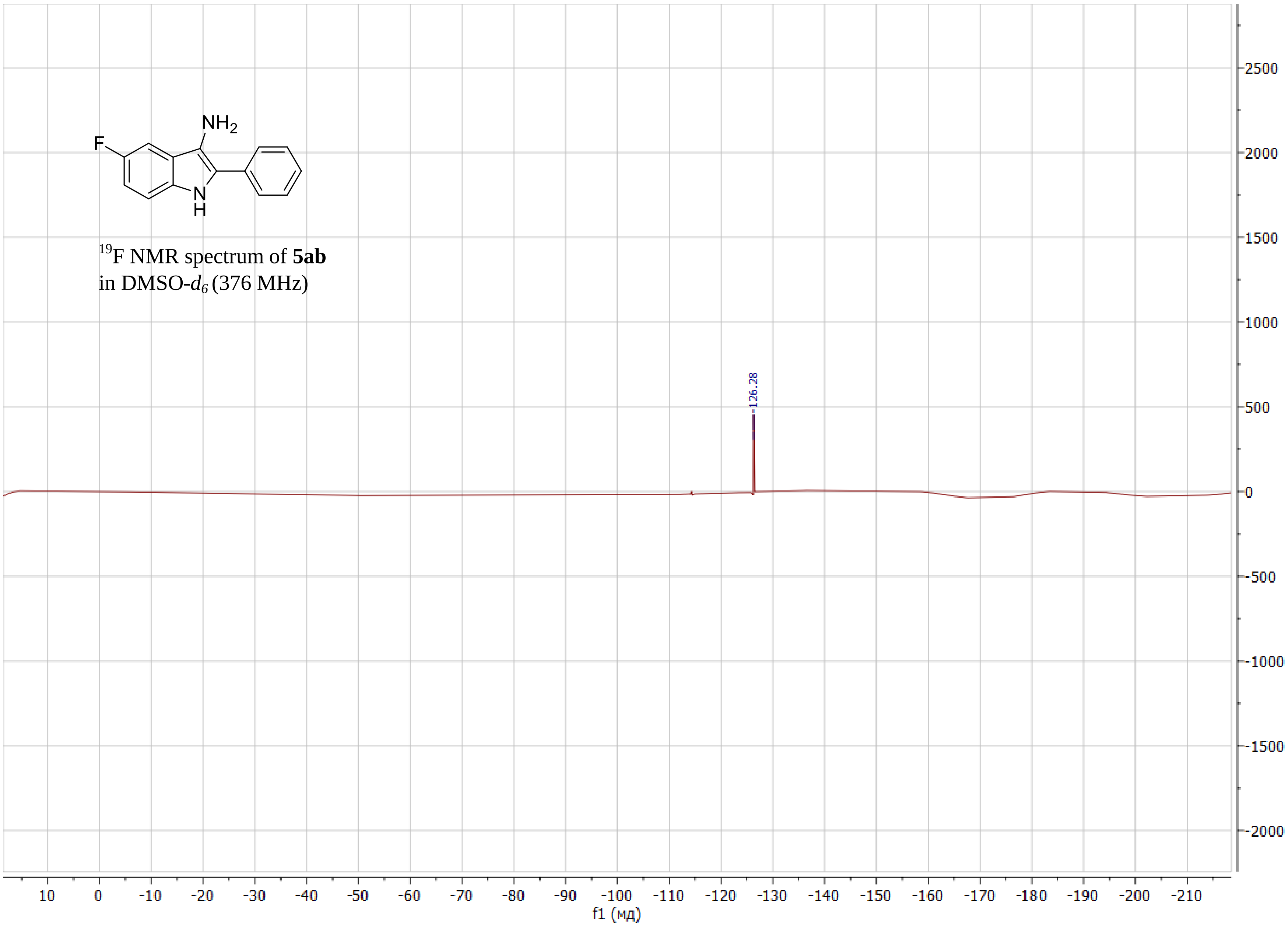

^{19}F NMR spectrum of **5ab** in DMSO-d_6 (376 MHz)

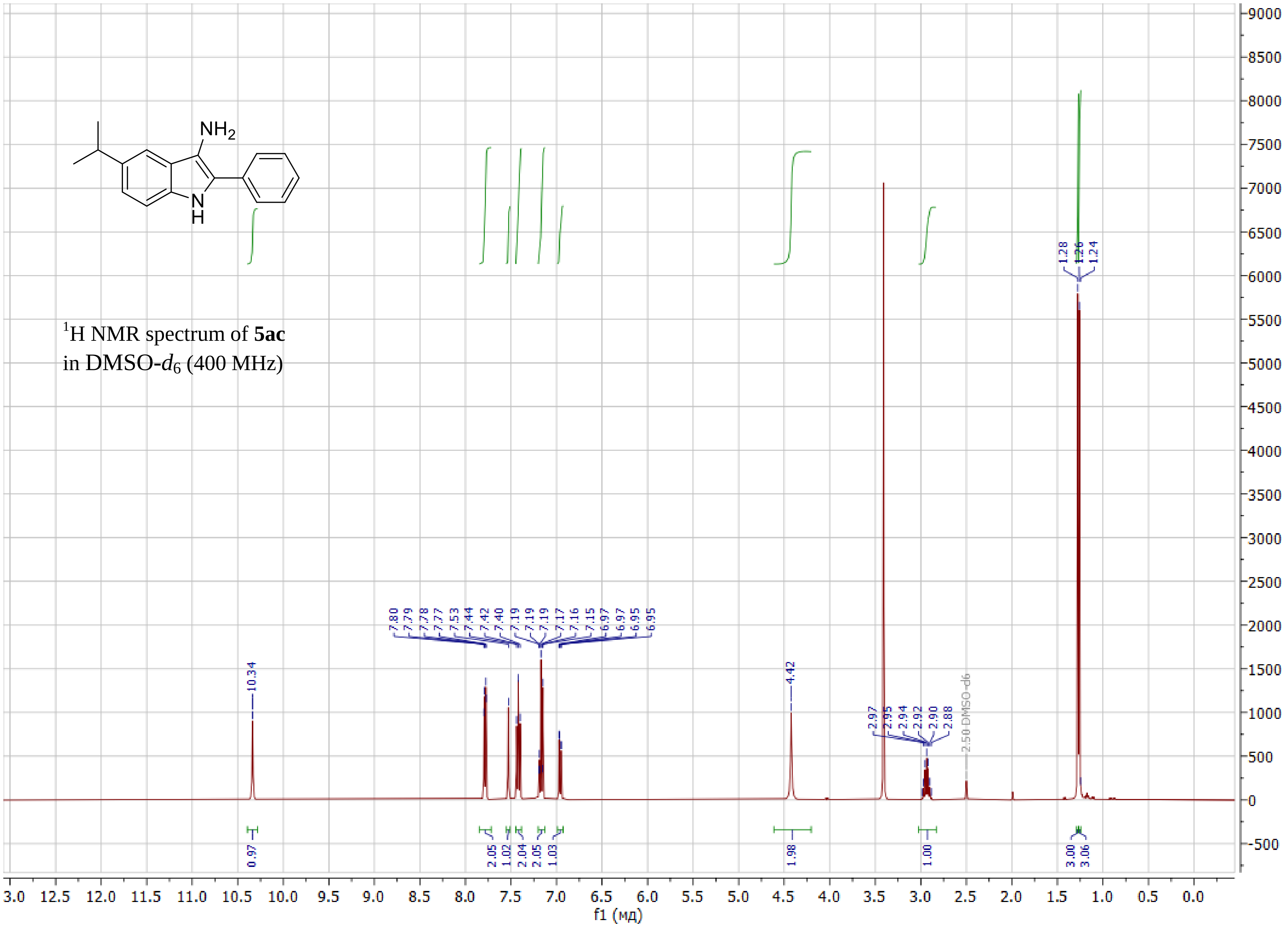

^{1}H NMR spectrum of **5ac** in DMSO-d_6 (400 MHz)

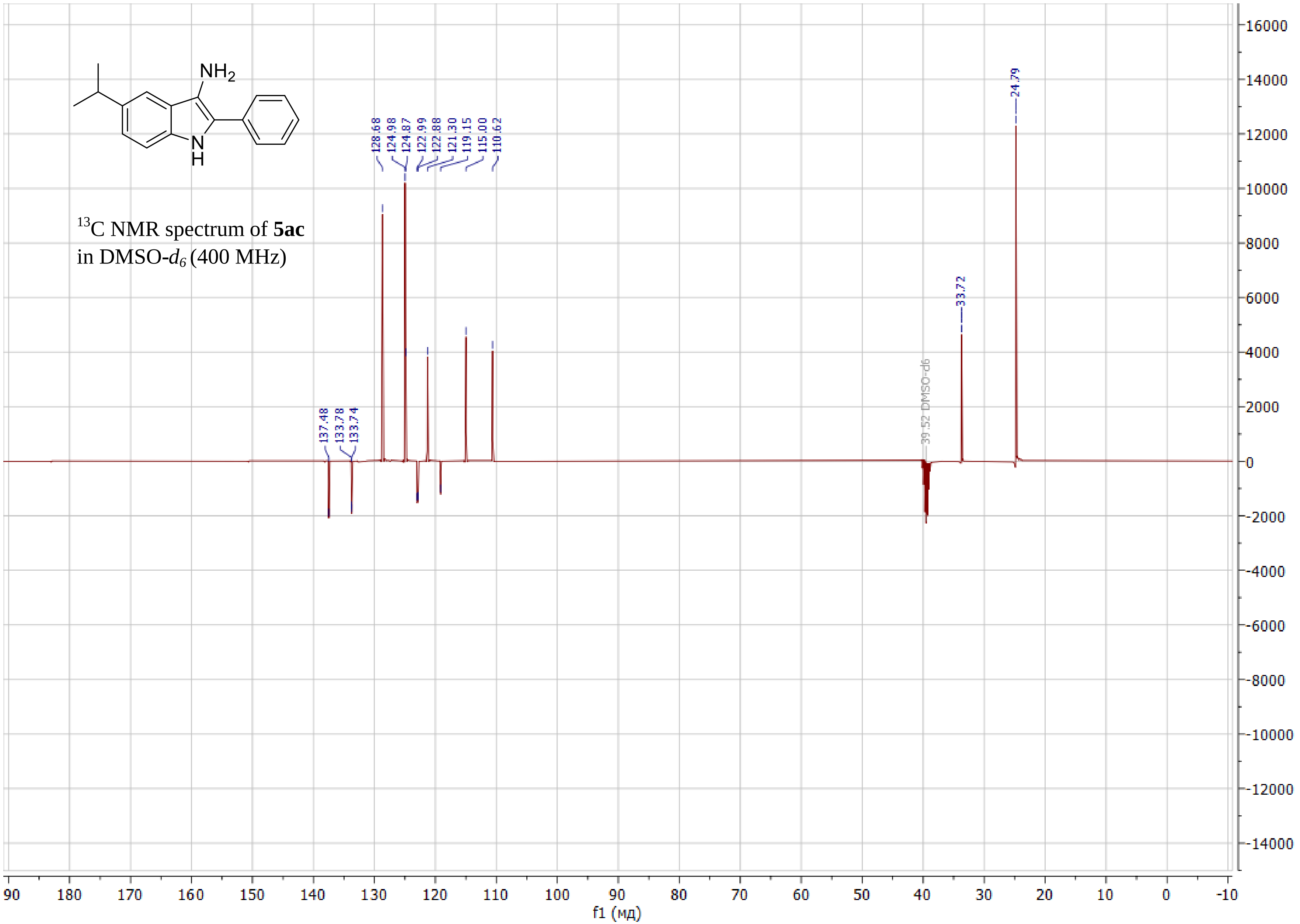
NH2
N
H
13C NMR spectrum of 5ac
in DMSO-d6 (400 MHz)
128.68
124.98
124.87
122.99
122.88
121.30
119.15
115.00
110.62
137.48
133.78
133.74
39.52 DMSO-d6
33.72
24.79
f1 (мд)

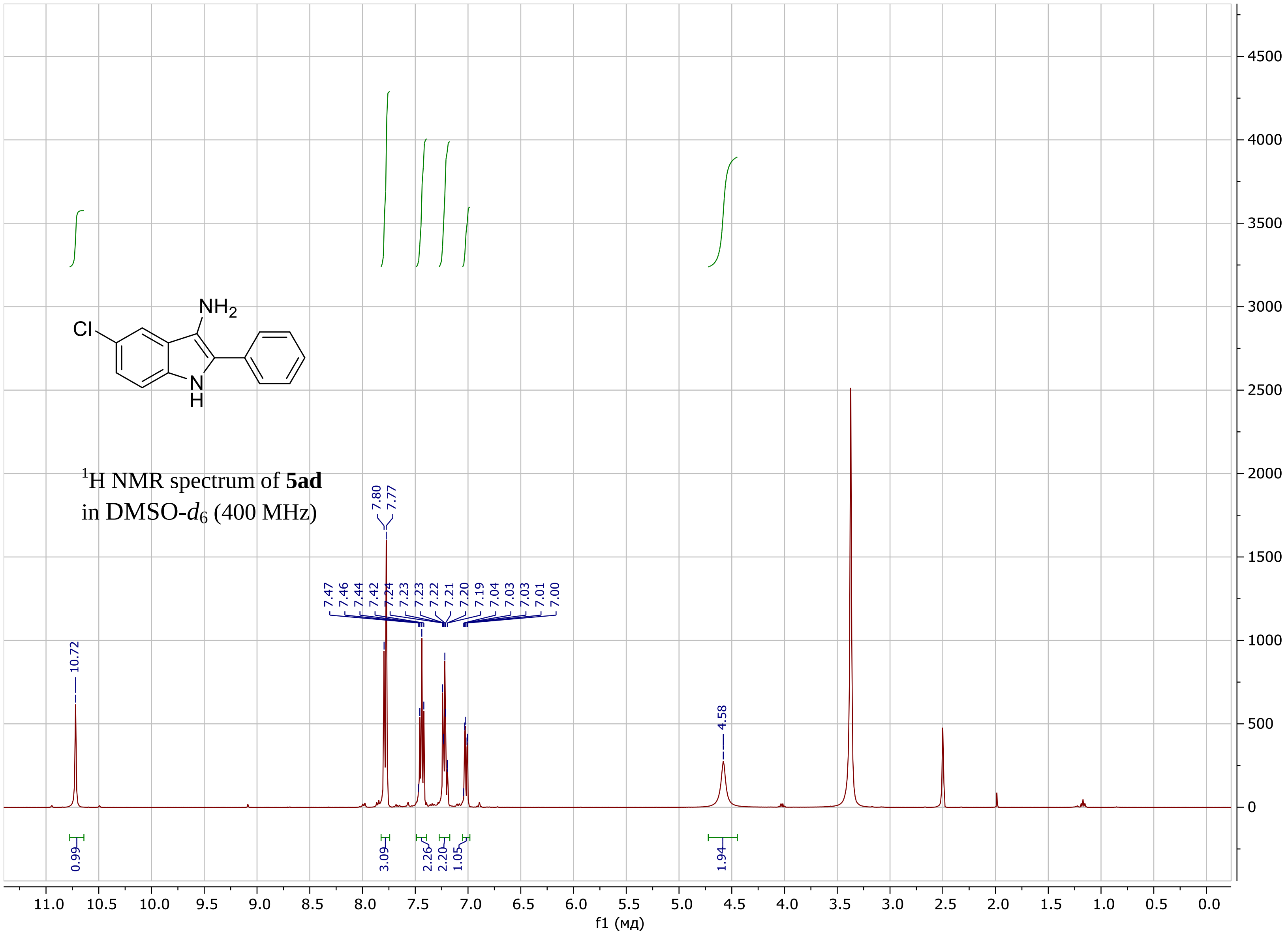

^{1}H NMR spectrum of **5ad** in DMSO-d_6 (400 MHz)

^{13}C NMR spectrum of **5ad** in DMSO-d_6 (101 MHz)

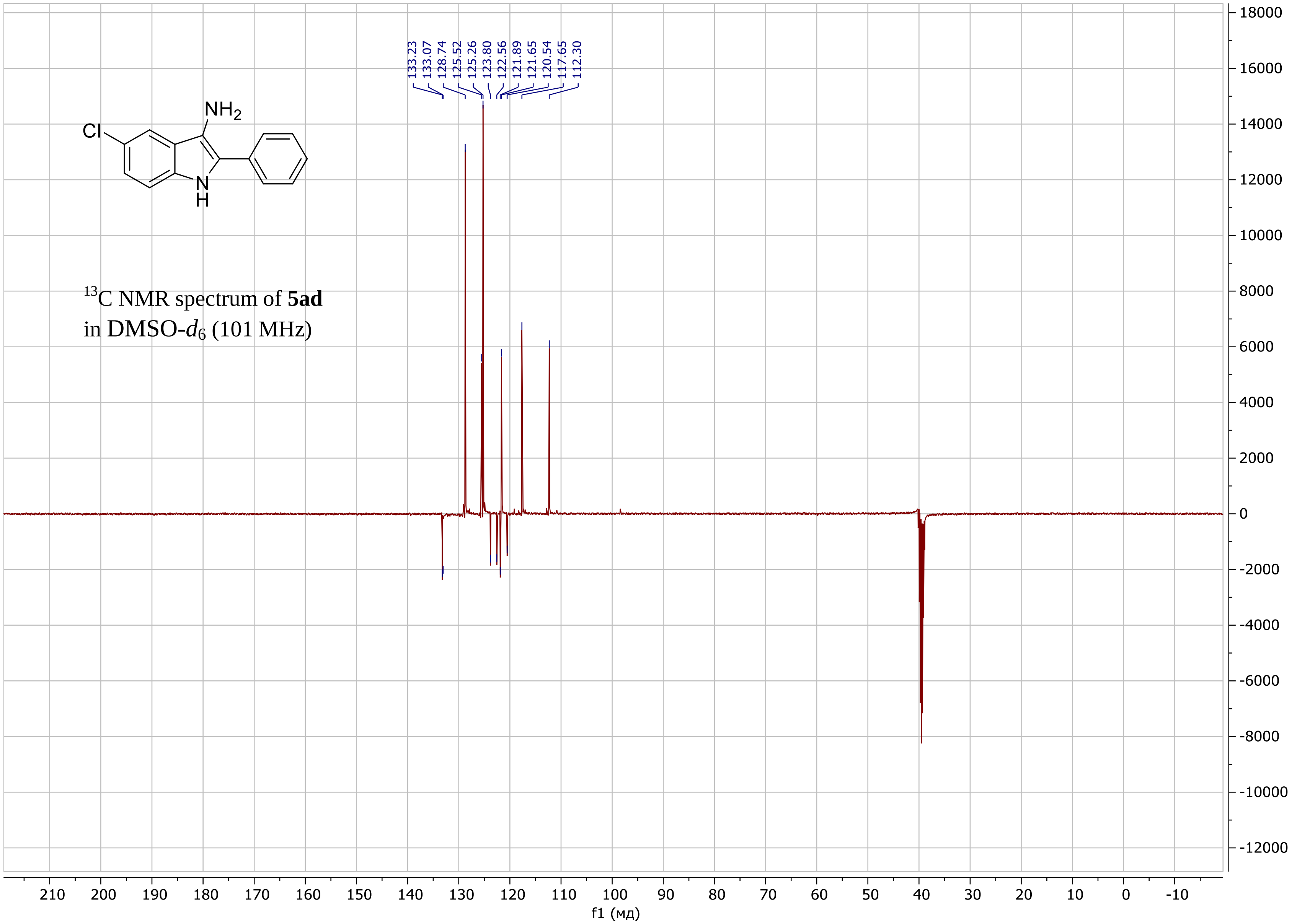

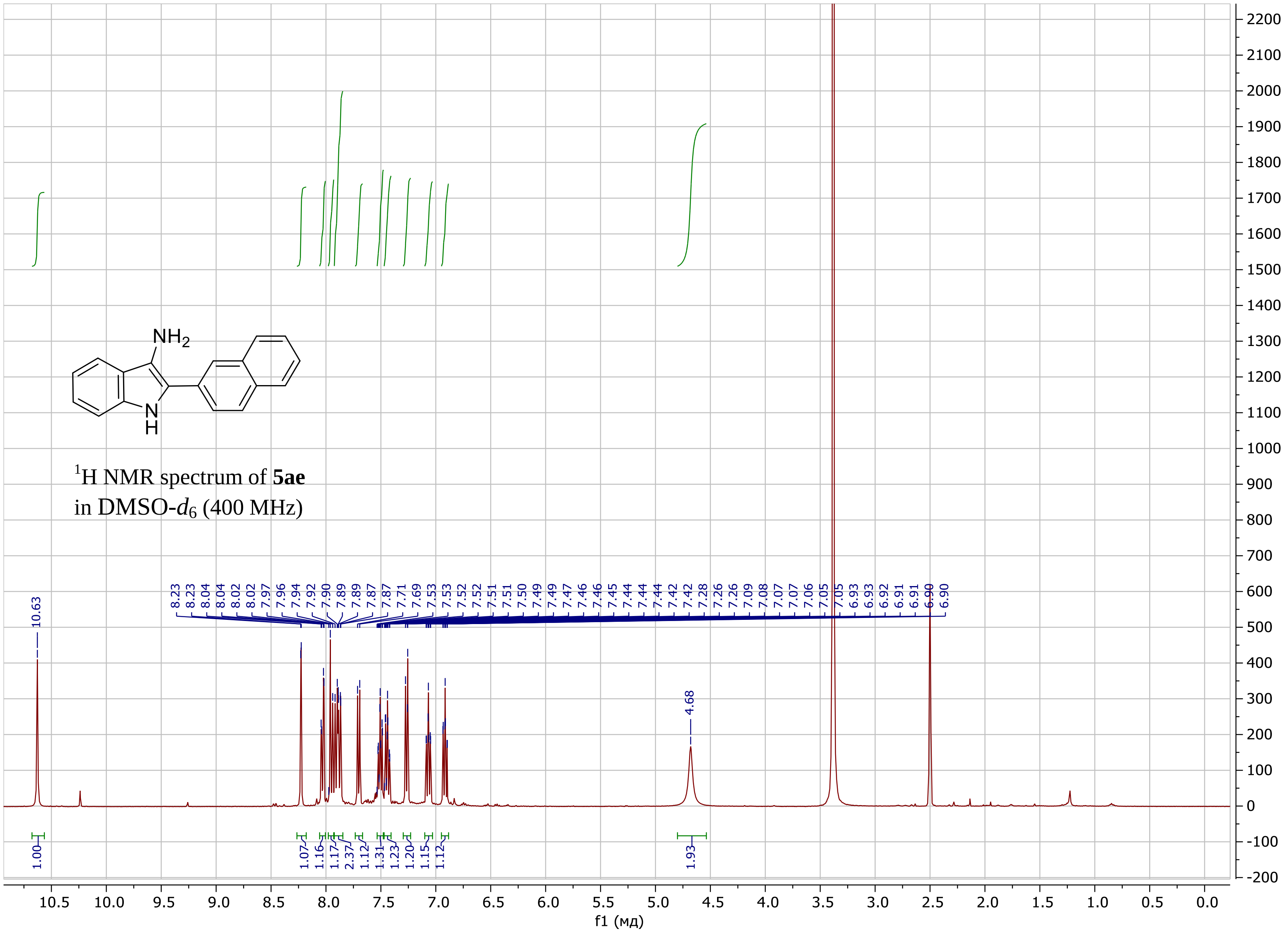

^{1}H NMR spectrum of **5ae** in DMSO-d_6 (400 MHz)

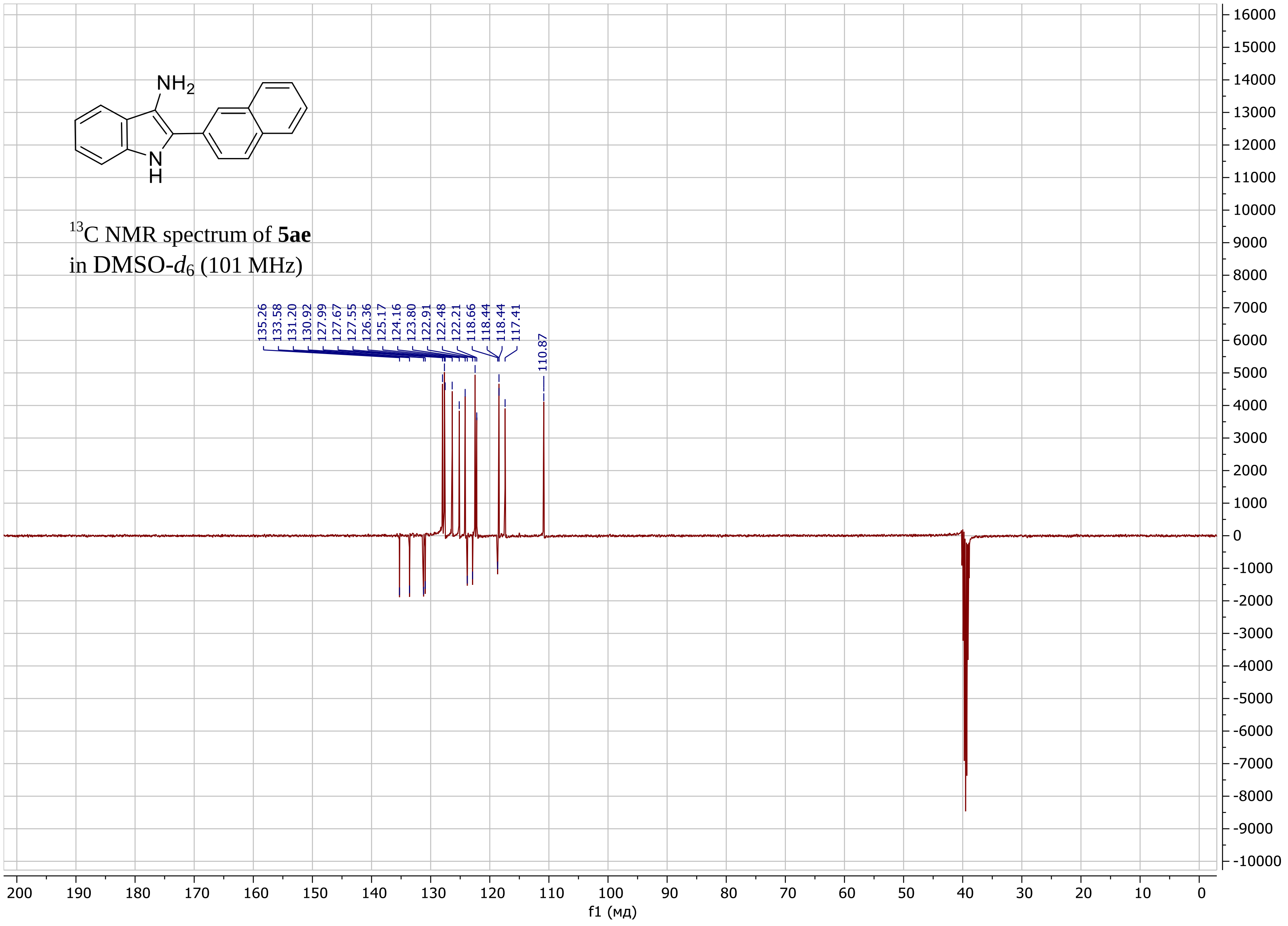

^{13}C NMR spectrum of **5ae** in DMSO-d_6 (101 MHz)

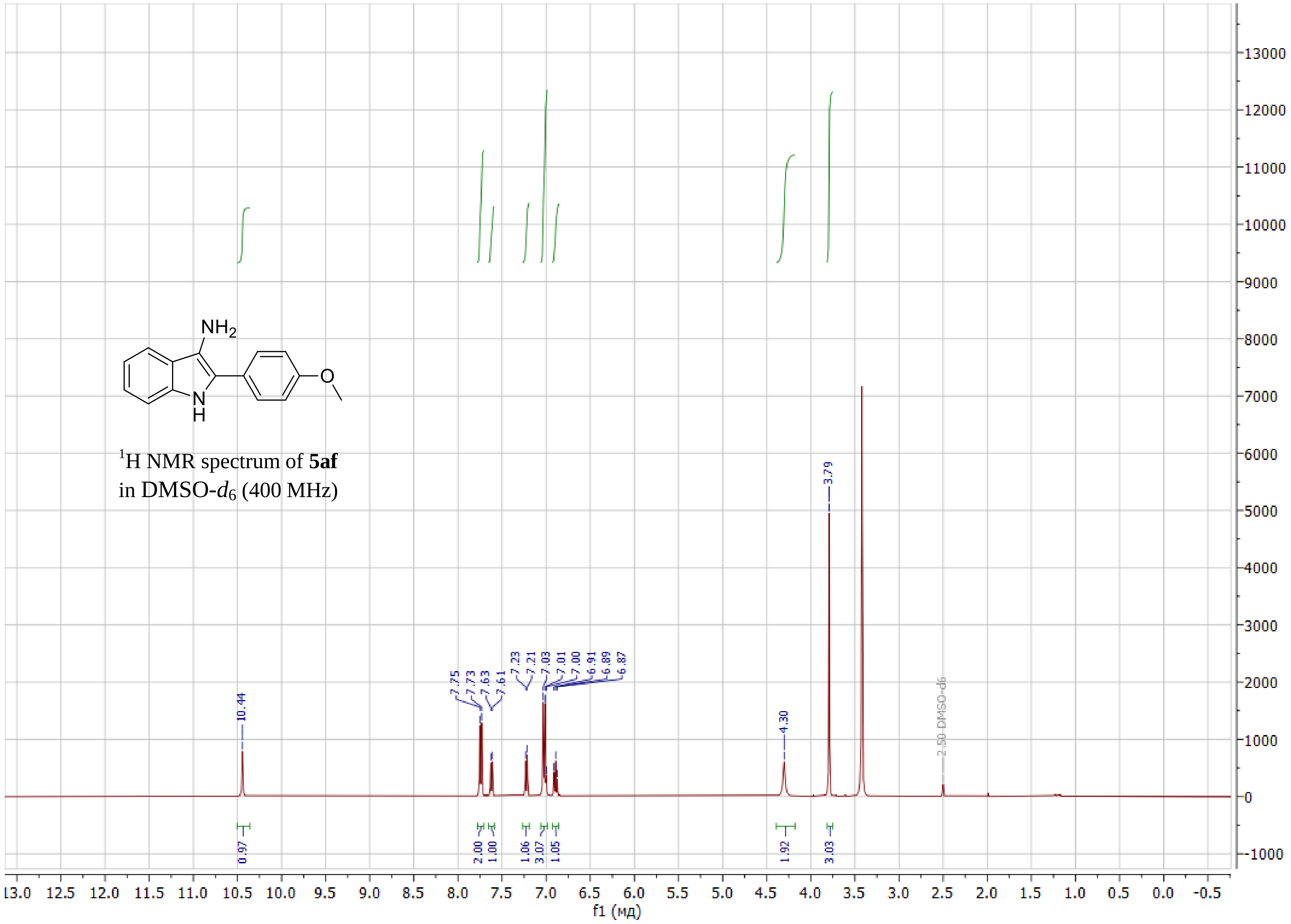

^{1}H NMR spectrum of **5af** in DMSO-d_6 (400 MHz)

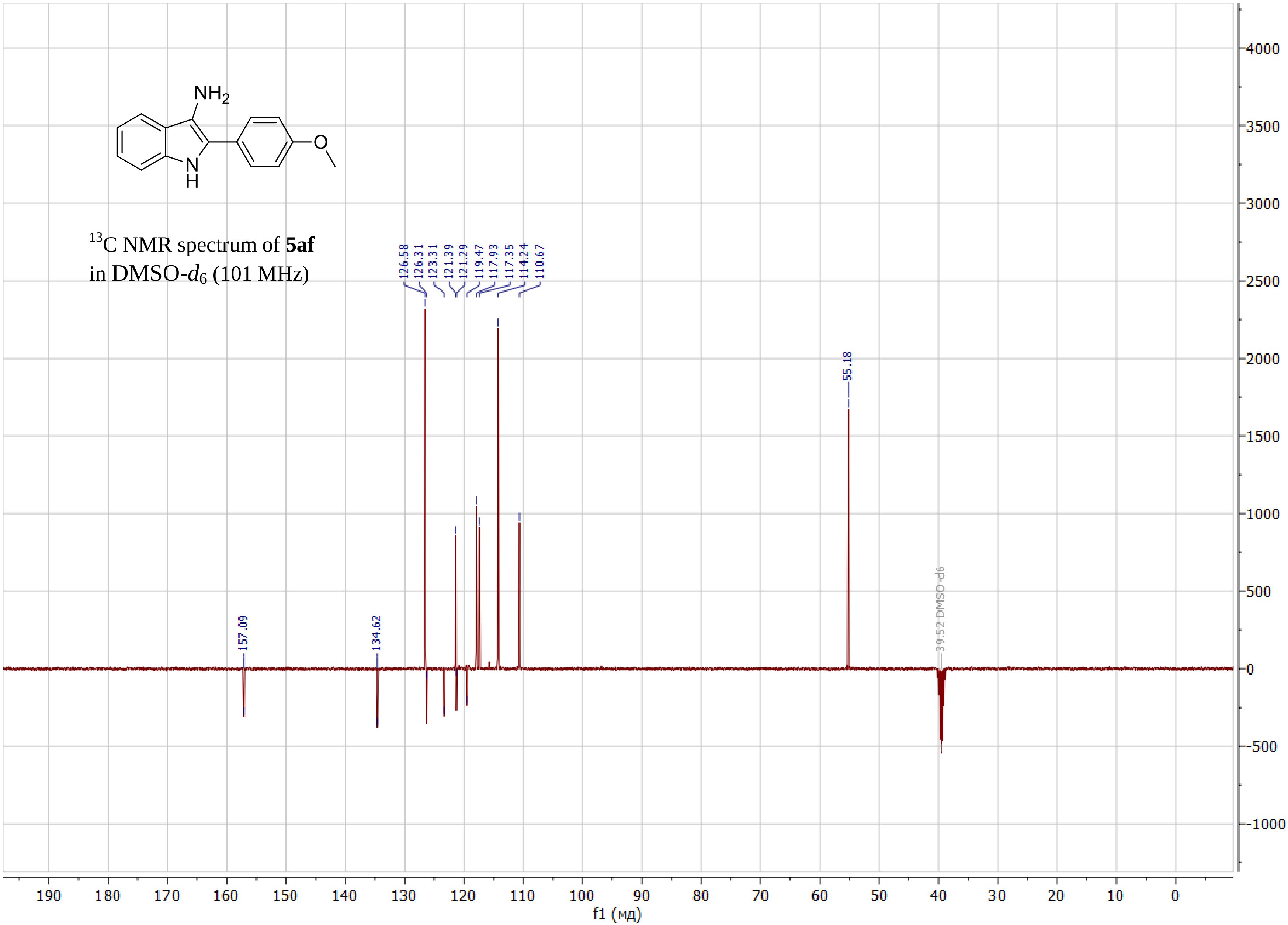

^{13}C NMR spectrum of **5af** in DMSO-d_6 (101 MHz)

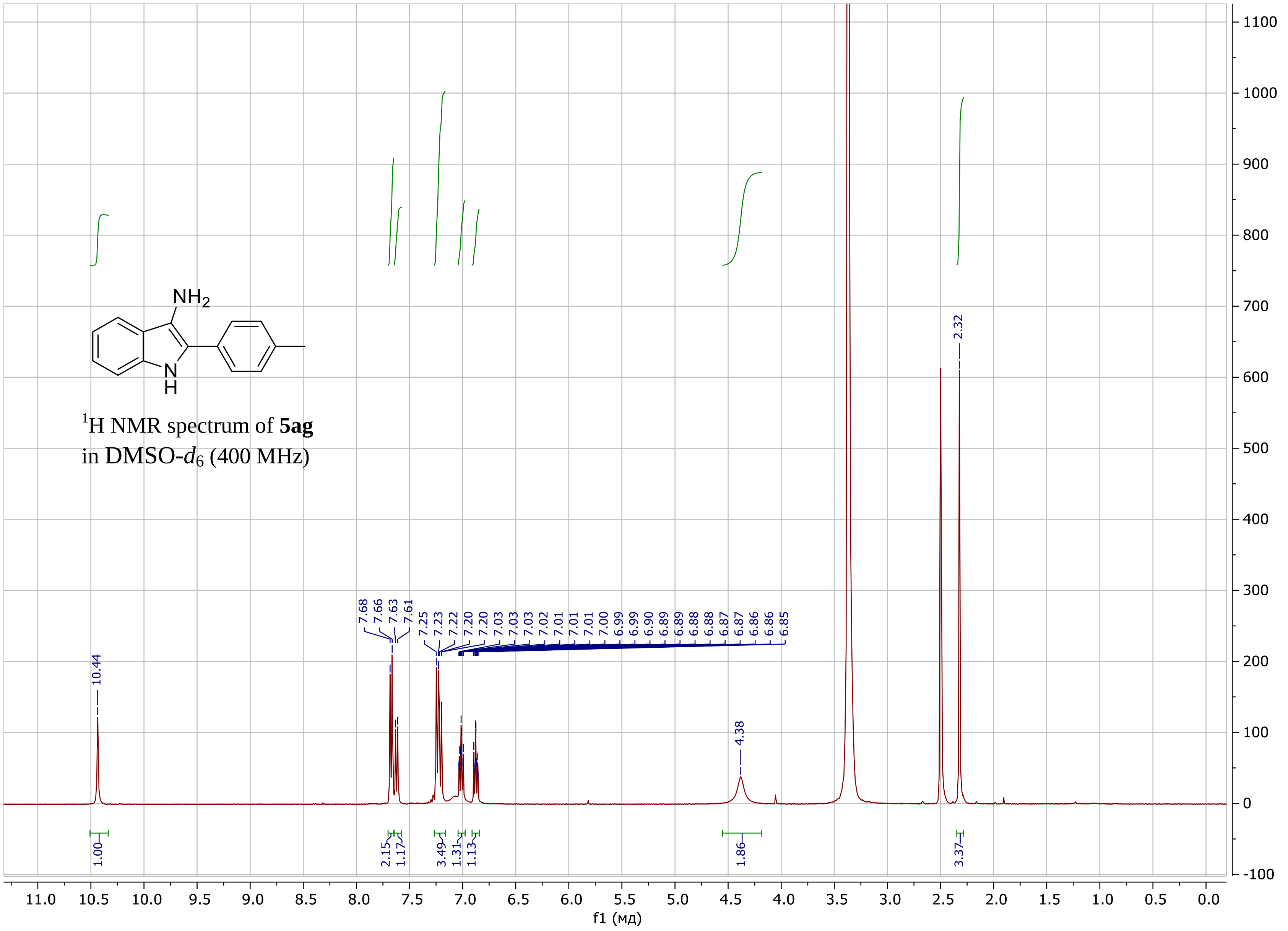

^{1}H NMR spectrum of **5ag** in DMSO-d_6 (400 MHz)

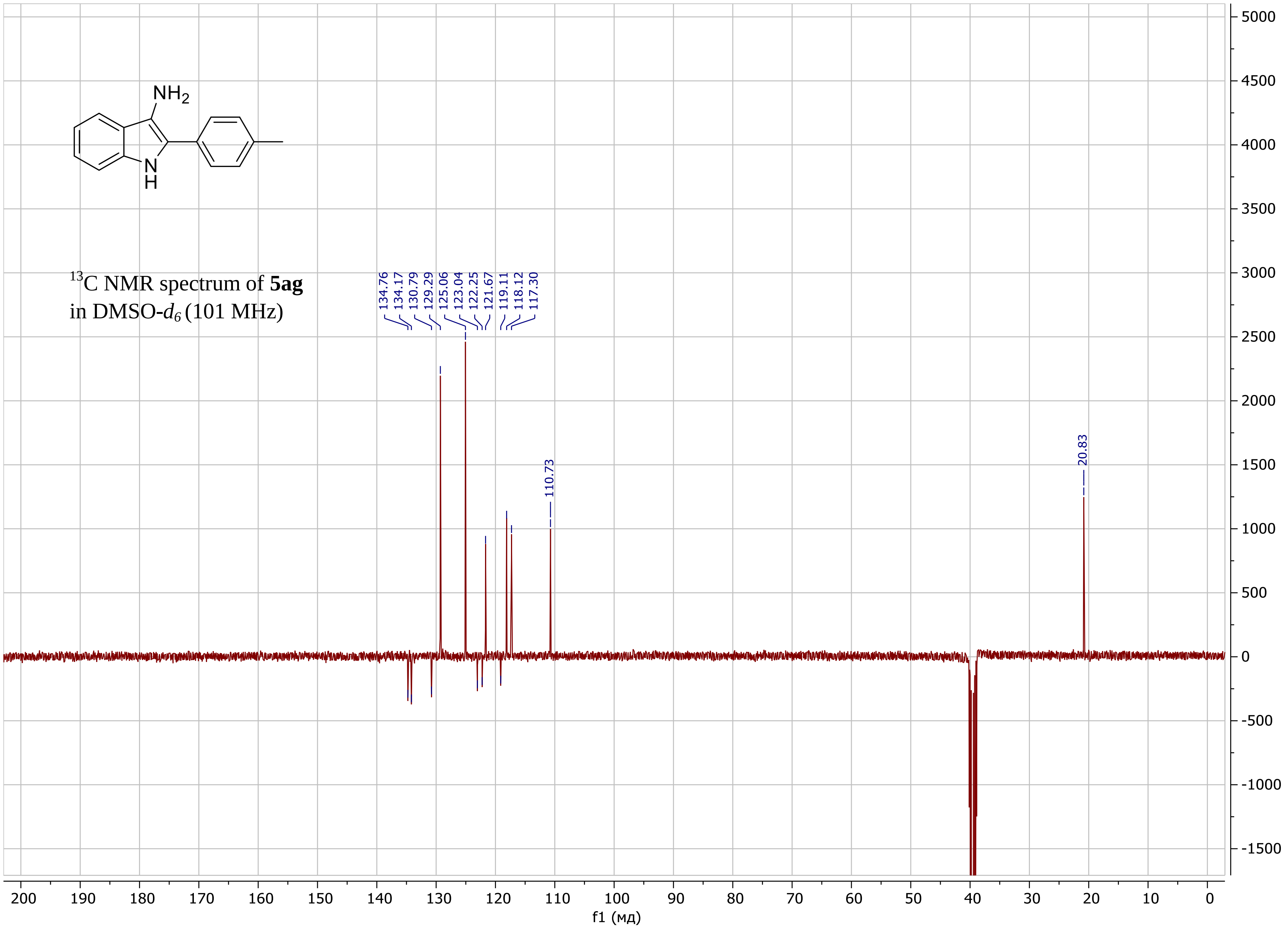

^{13}C NMR spectrum of **5ag** in DMSO-d_6 (101 MHz)

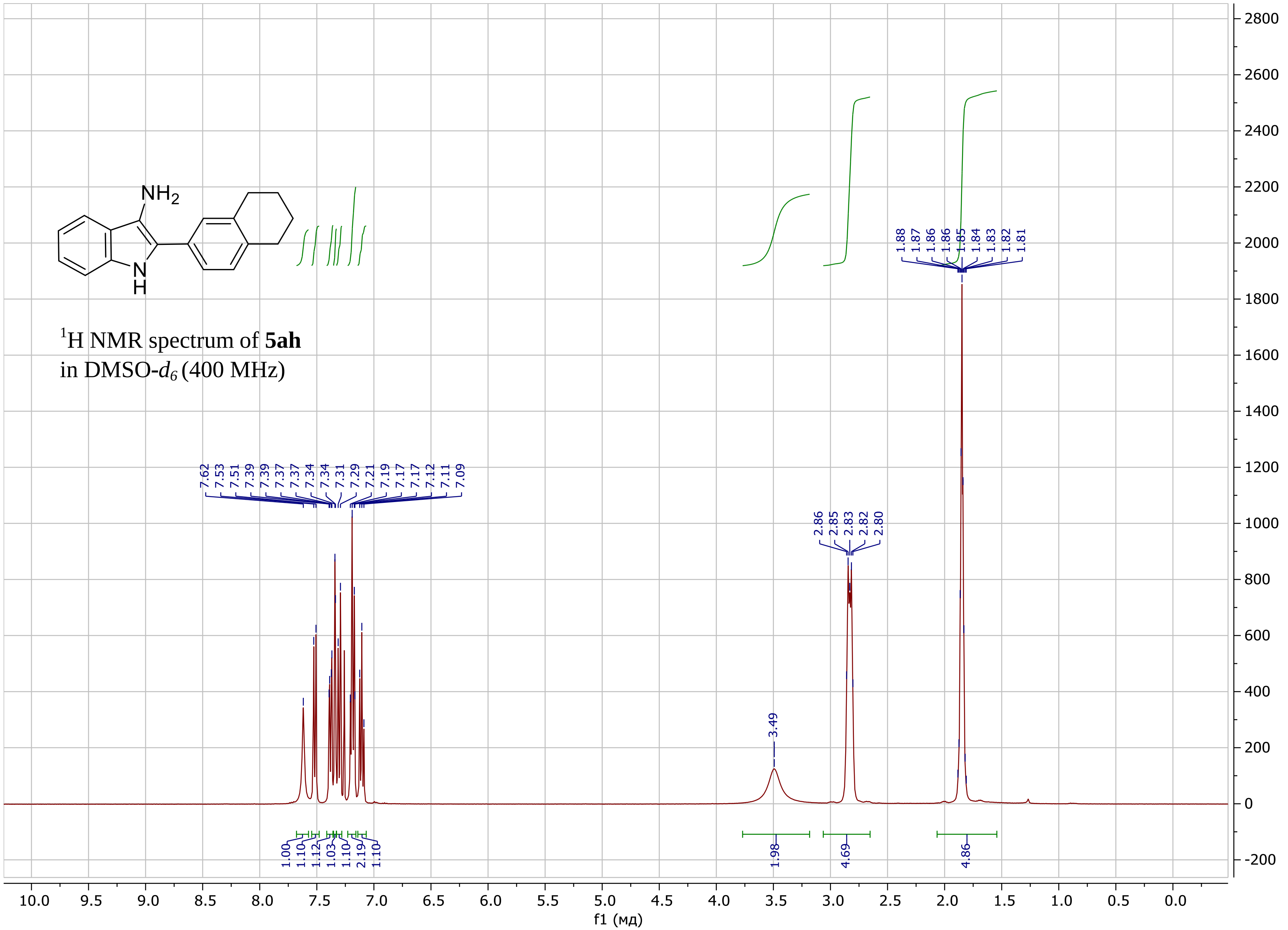

NH2
N
H
^{1}H NMR spectrum of 5ah
in DMSO-d6 (400 MHz)
f1 (мд)

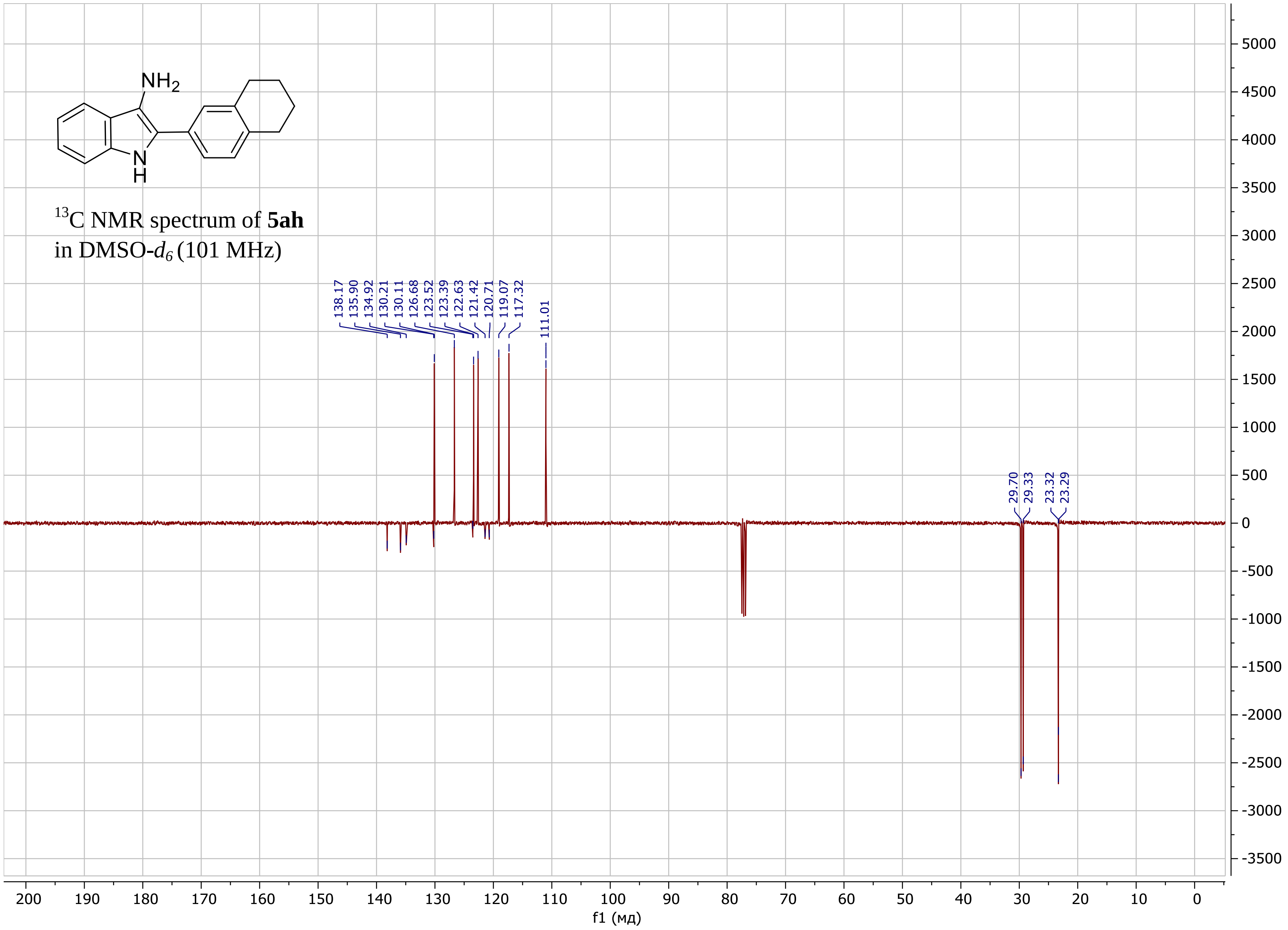
NH2
N
H
13C NMR spectrum of 5ah
in DMSO-d6 (101 MHz)
138.17
135.90
134.92
130.21
130.11
126.68
123.52
123.39
122.63
121.42
120.71
119.07
117.32
111.01
29.70
29.33
23.32
23.29
f1 (мд)

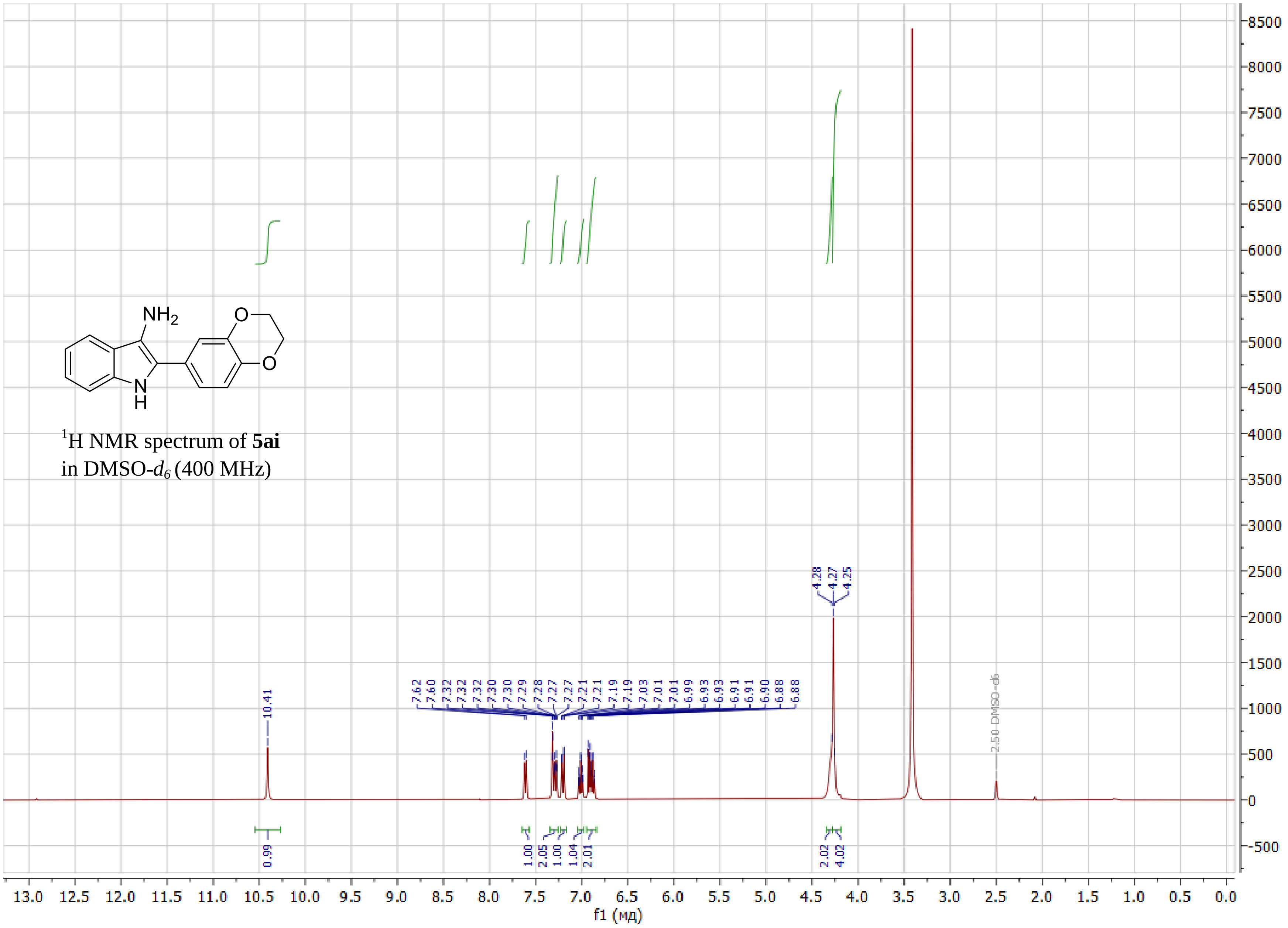

^{1}H NMR spectrum of **5ai** in DMSO-d_6 (400 MHz)

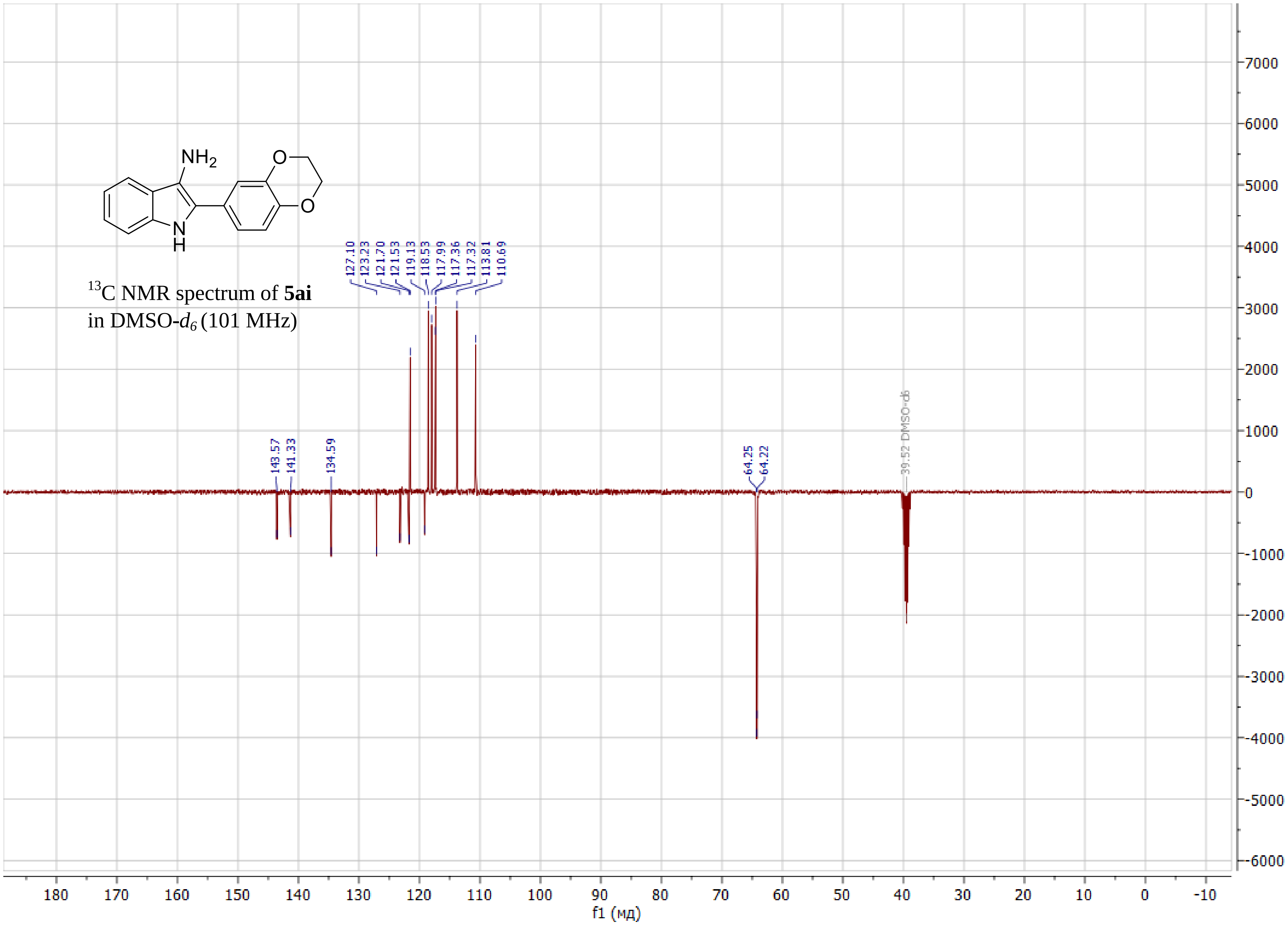

^{13}C NMR spectrum of **5ai** in DMSO-d_6 (101 MHz)

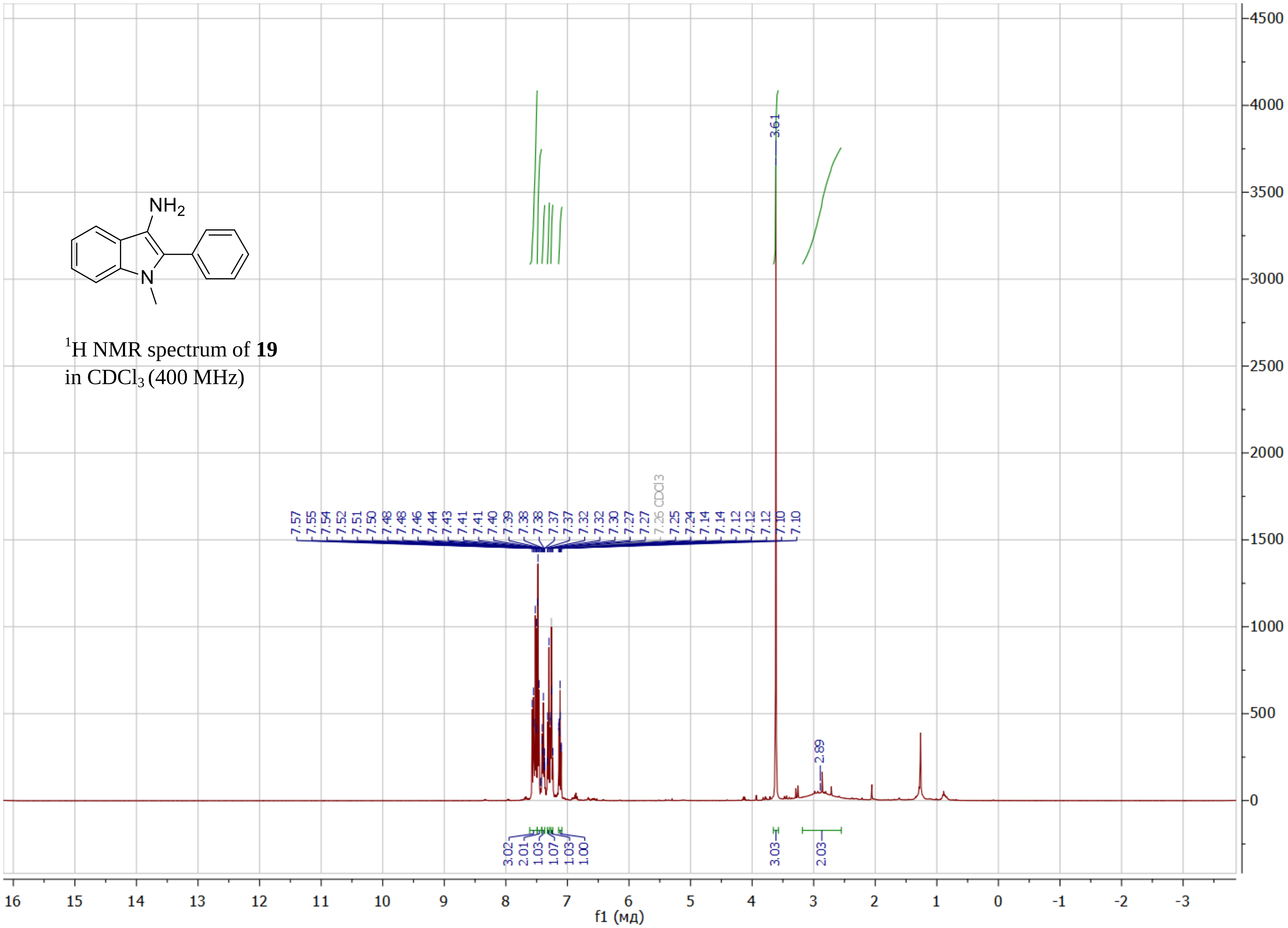

^{1}H NMR spectrum of **19**
in $CDCl_3$ (400 MHz)

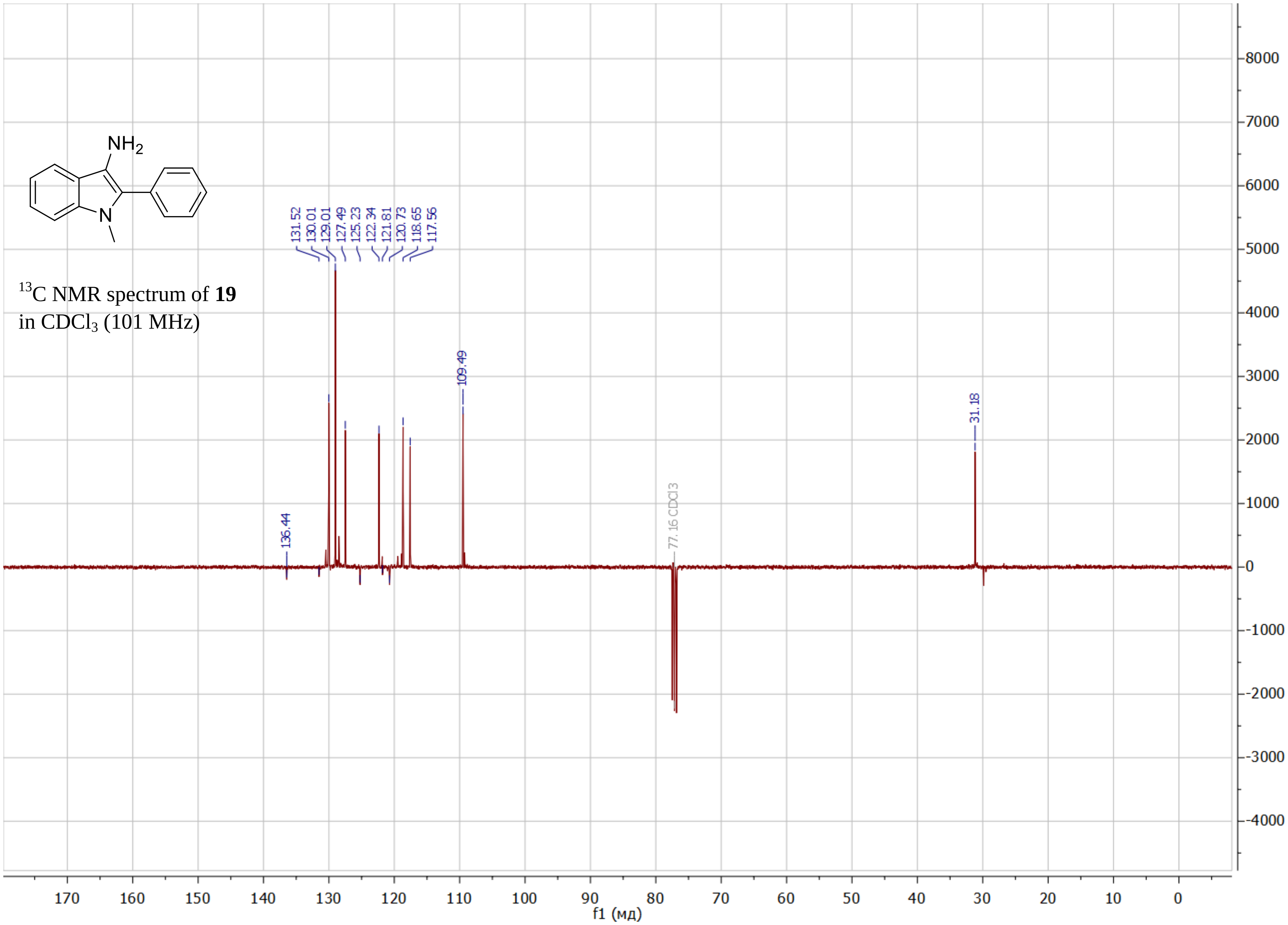

^{13}C NMR spectrum of **19** in $CDCl_3$ (101 MHz)

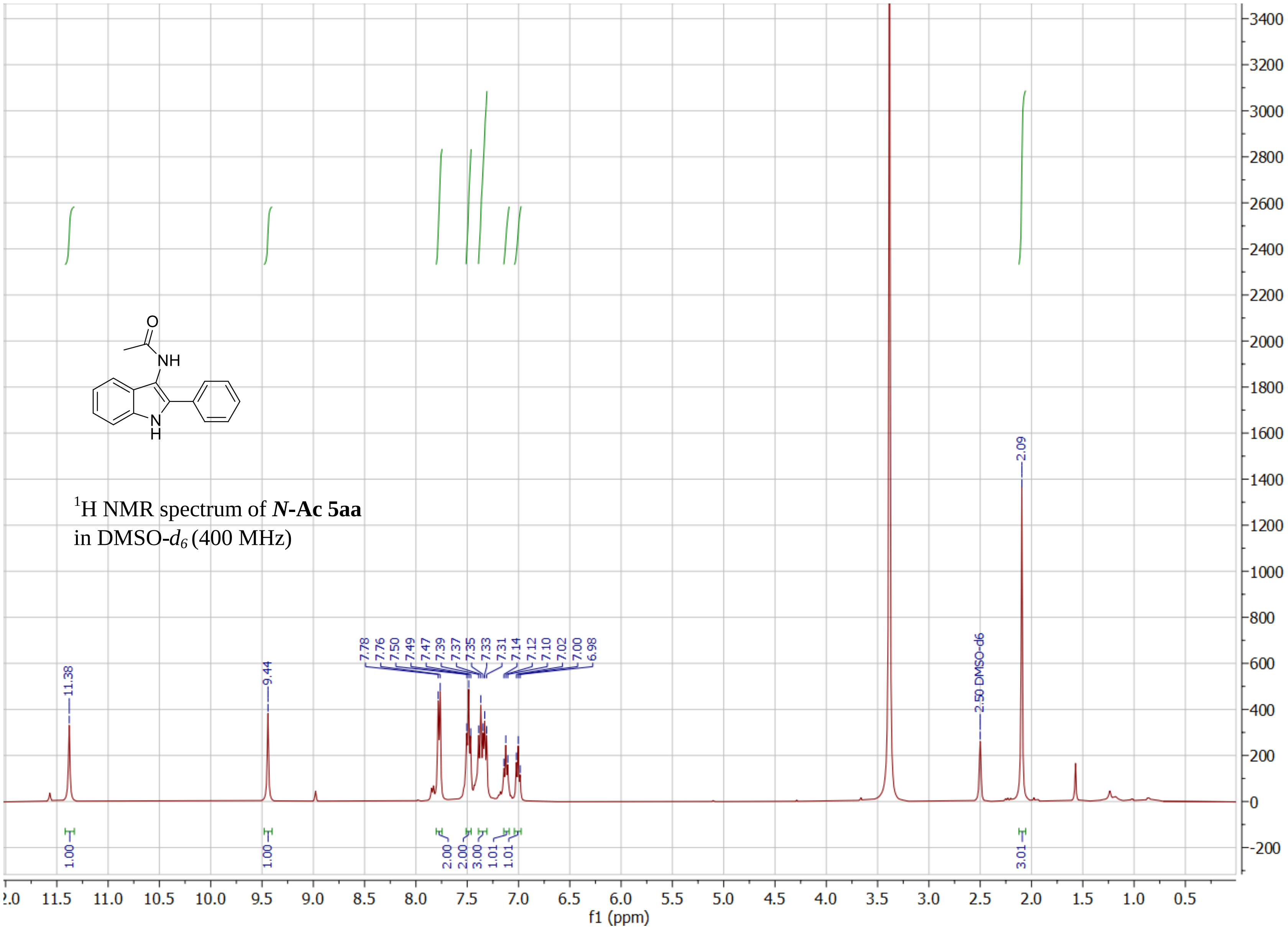

^{1}H NMR spectrum of ***N*-Ac 5aa**
in DMSO-d_6 (400 MHz)

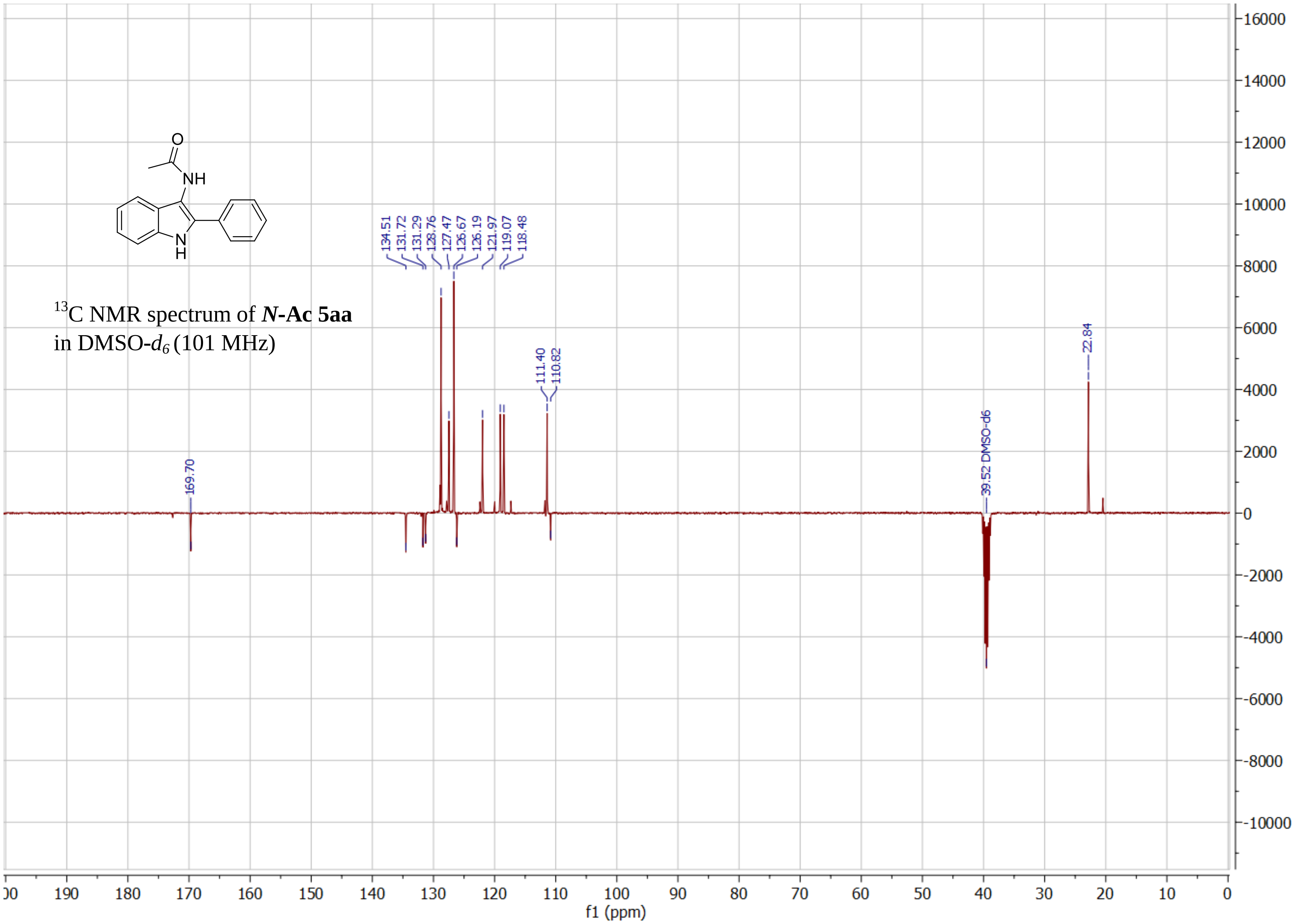

^{13}C NMR spectrum of ***N*-Ac 5aa**
in DMSO-d_6 (101 MHz)

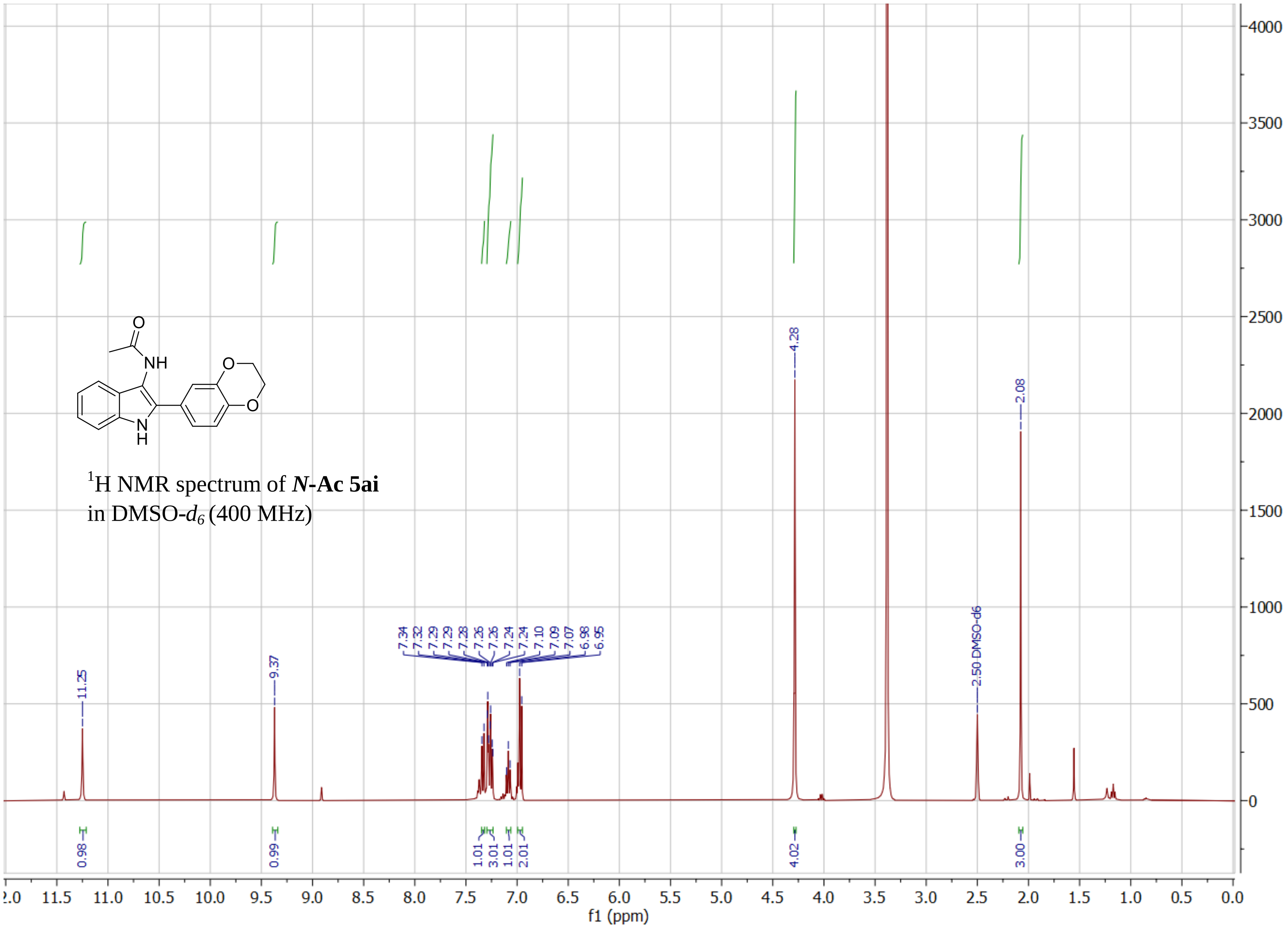

^{1}H NMR spectrum of ***N*-Ac 5ai**
in DMSO-d_6 (400 MHz)

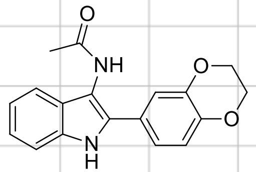

^{13}C NMR spectrum of ***N*-Ac 5ai**
in DMSO-d_6 (101 MHz)

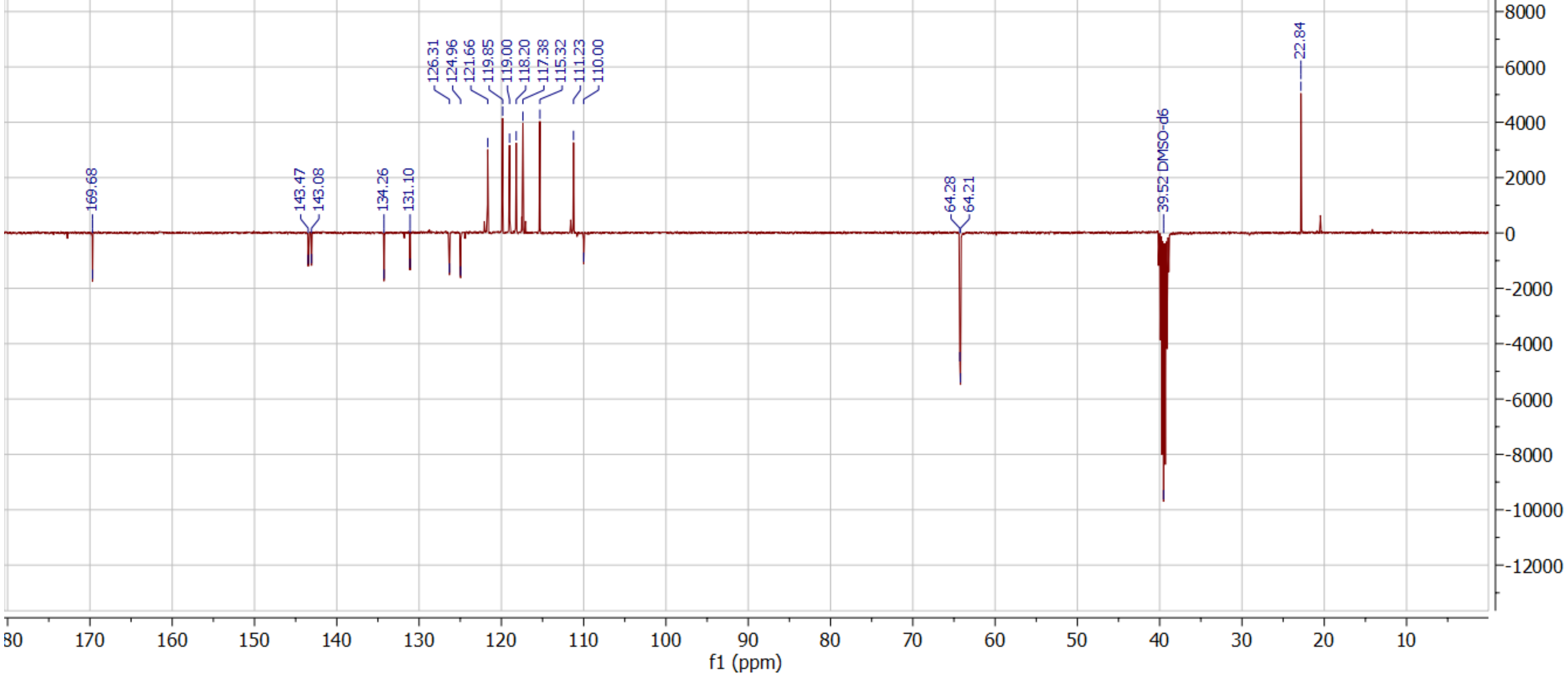

^{1}H and ^{13}C NMR spectral charts for 1-methyl-2-phenyl-1*H*-indole **J**

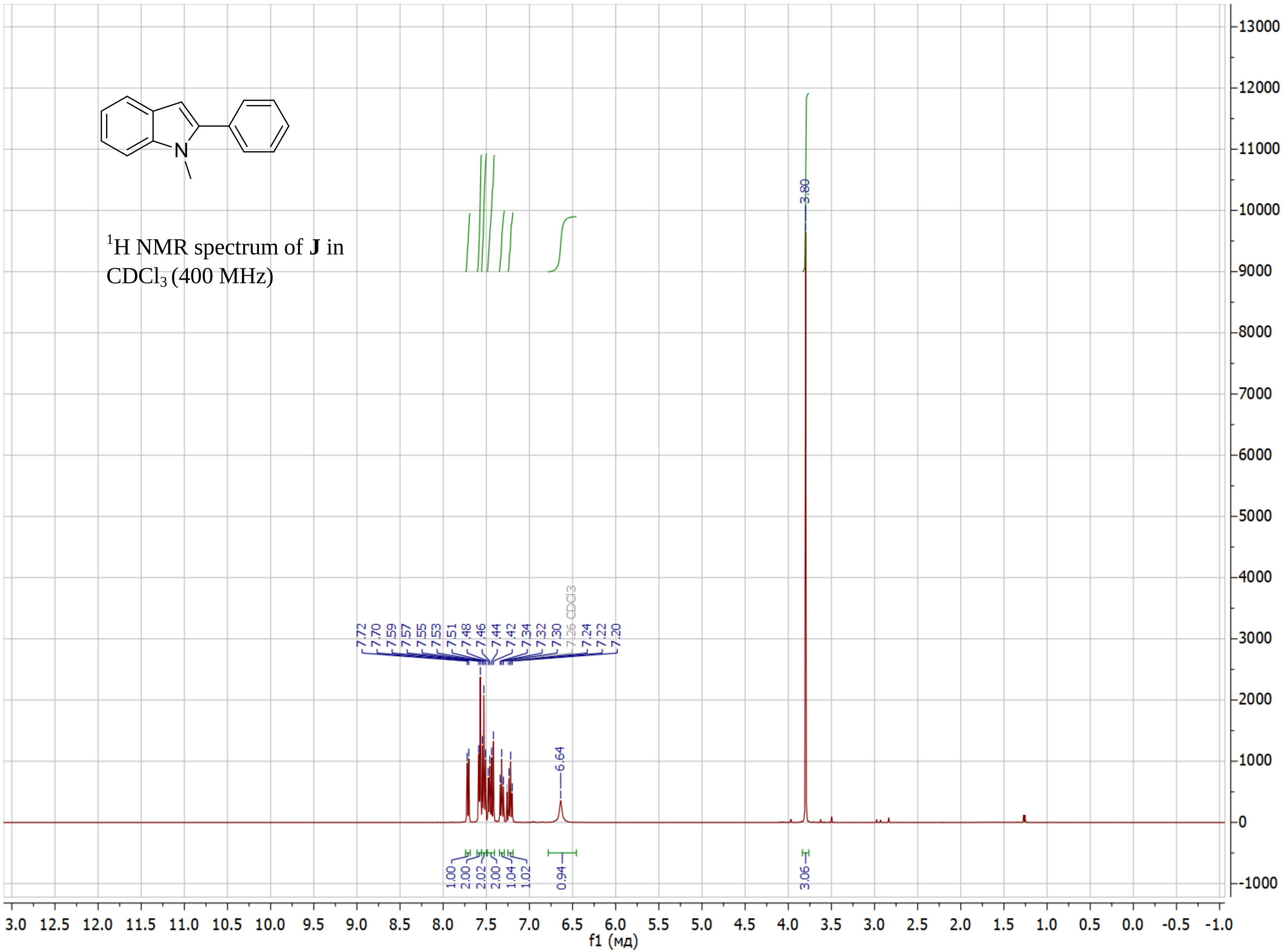

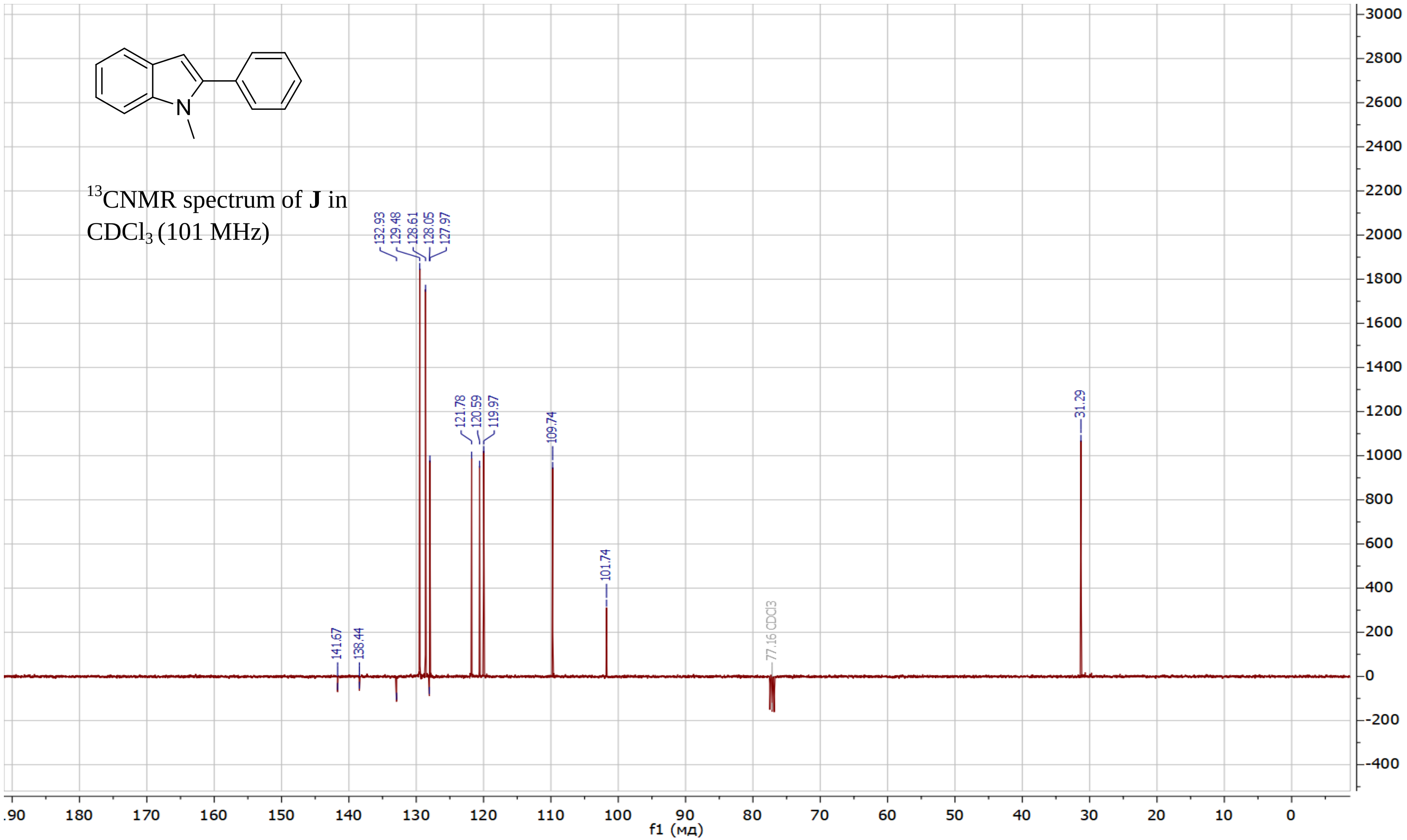

^{13}CNMR spectrum of **J** in $CDCl_3$ (101 MHz)

HRMS spectral chart for **4ab**, **4ad, 5aa-ai**, **19**, ***N*-Ac 5aa** and ***N*-Ac 5ai**

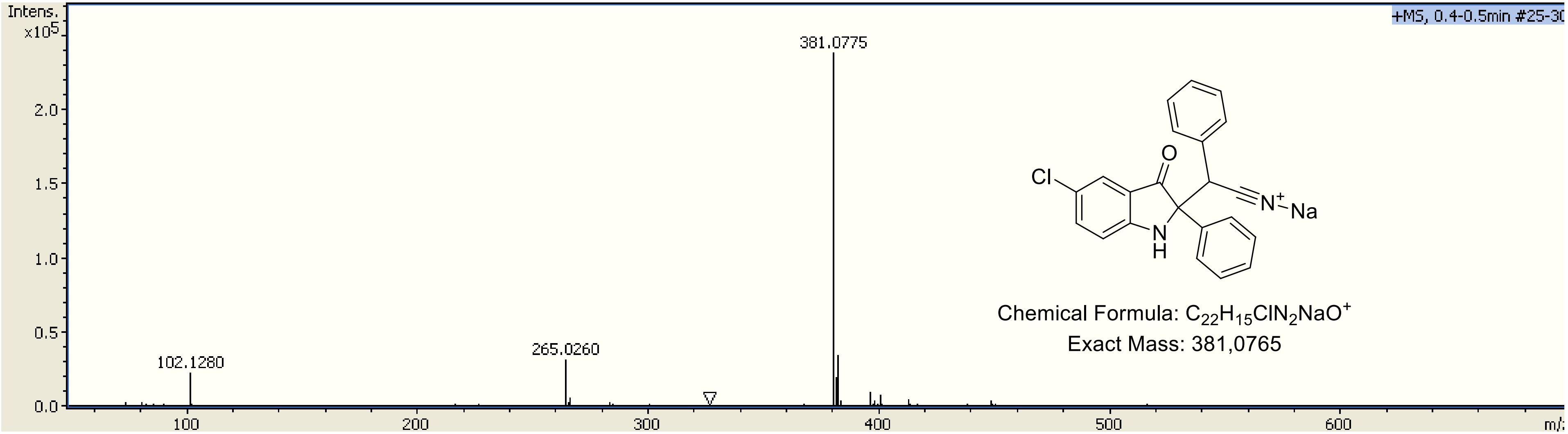

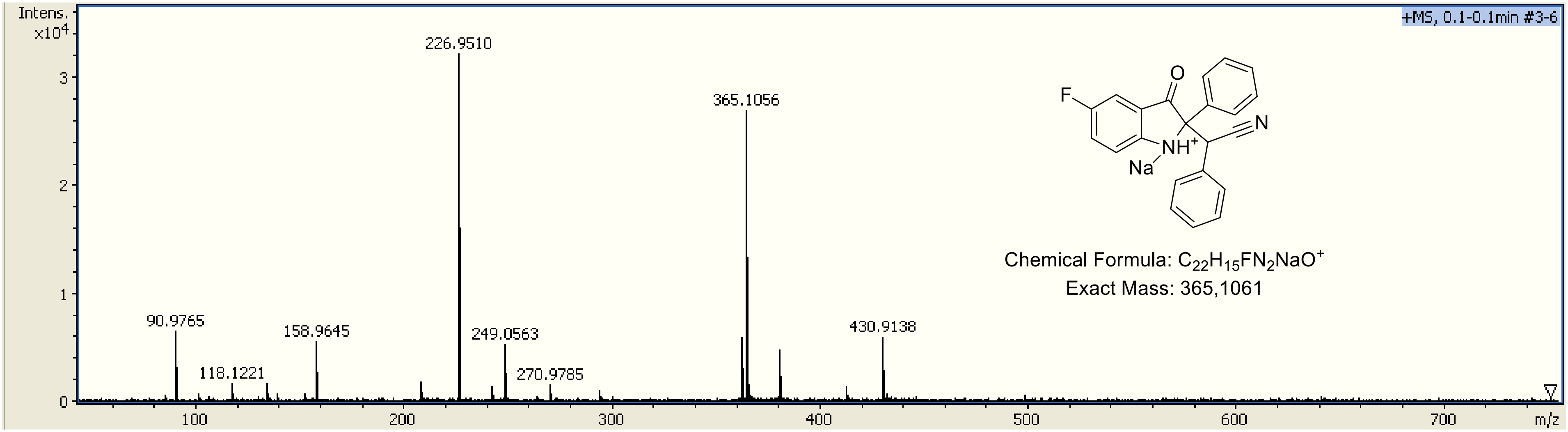

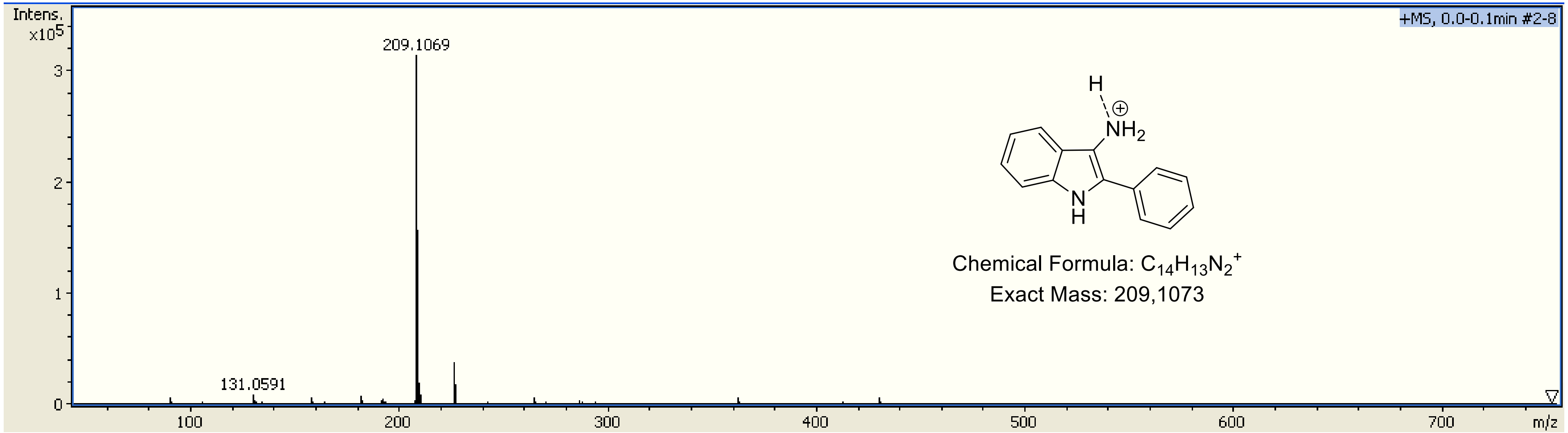
Intens.
x10^5
+MS, 0.0-0.1min #2-8
209.1069
131.0591
m/z
H
NH2
N
H
Chemical Formula: C14H13N2+
Exact Mass: 209,1073

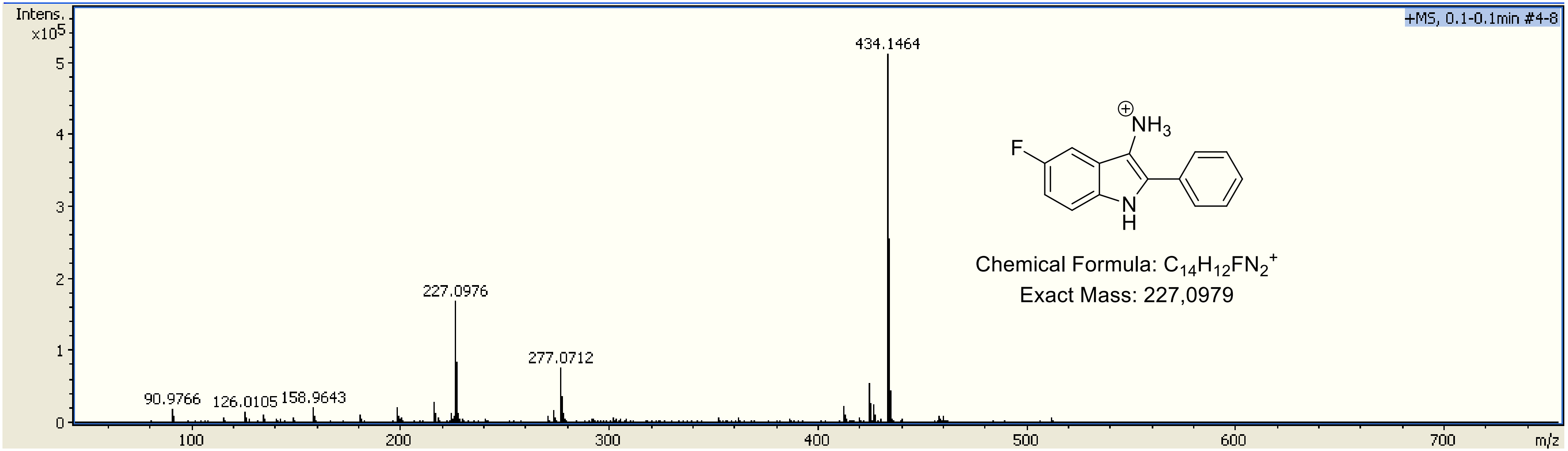
Intens.
x10^5
+MS, 0.1-0.1min #4-8
434.1464
227.0976
277.0712
90.9766
126.0105
158.9643
m/z
NH3
F
N
H
Chemical Formula: C14H12FN2+
Exact Mass: 227,0979

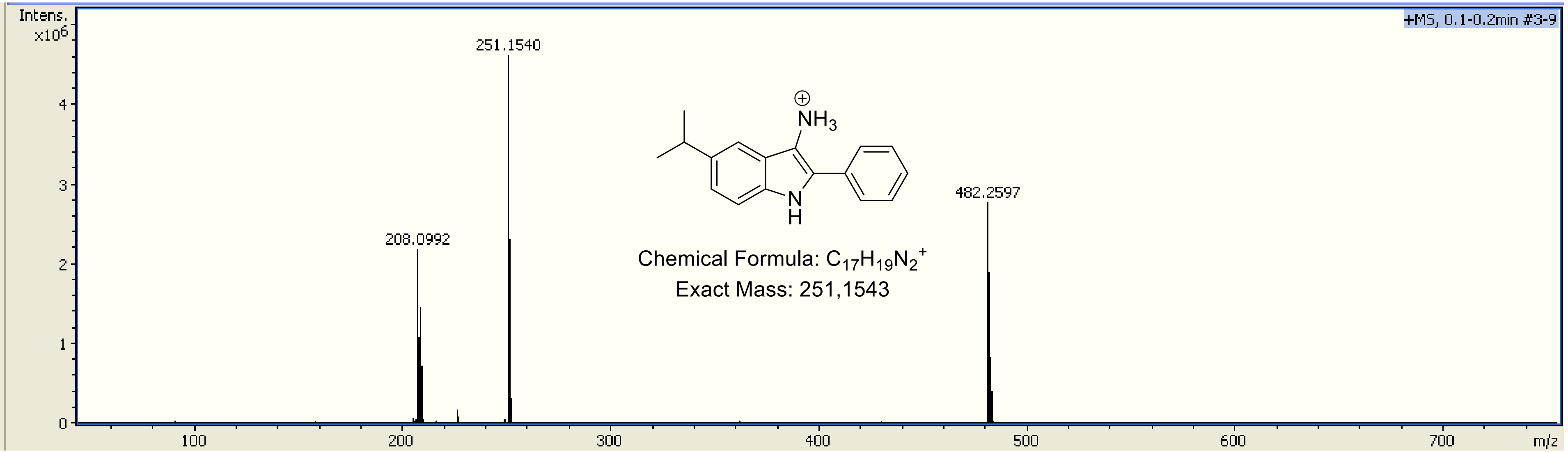

Intens.
x10^6
+MS, 0.1-0.2min #3-9
251.1540
208.0992
482.2597
NH3
N
H
Chemical Formula: $C_{17}H_{19}N_2^+$
Exact Mass: 251,1543
m/z

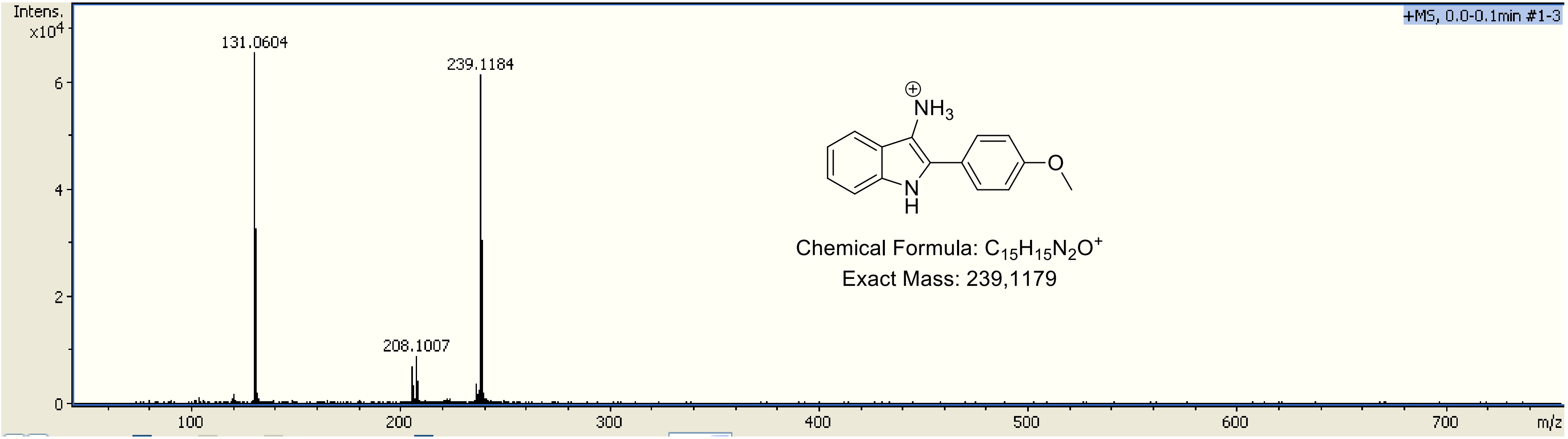

Intens.
x10^4
+MS, 0.0-0.1min #1-3
131.0604
239.1184
208.1007
NH3
N
H
O
Chemical Formula: $C_{15}H_{15}N_2O^+$
Exact Mass: 239,1179
m/z

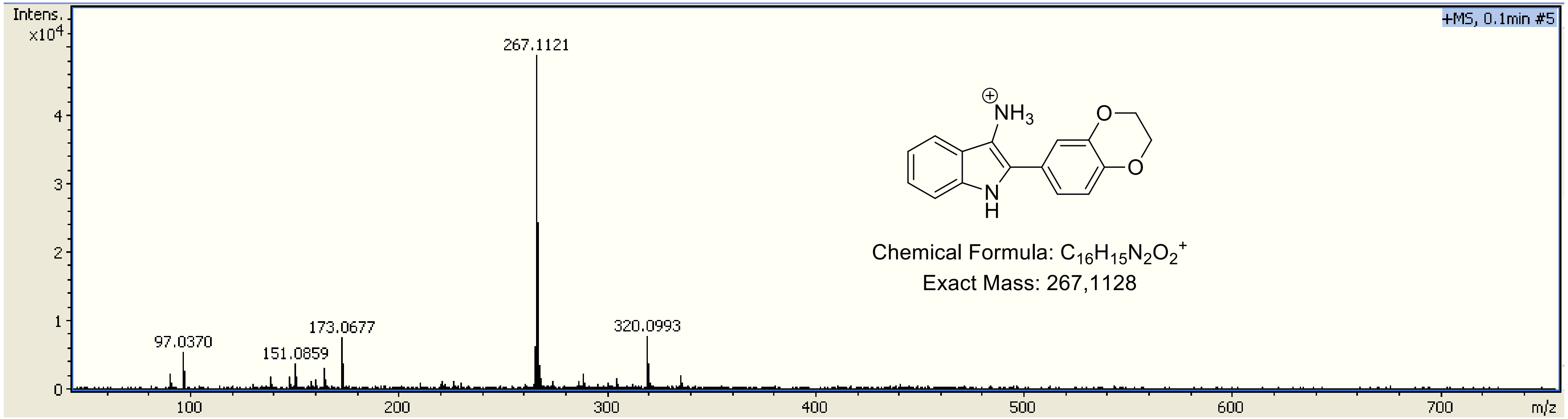
Intens.
x10^4
+MS, 0.1min #5
267.1121
320.0993
173.0677
151.0859
97.0370
m/z
Chemical Formula: $C_{16}H_{15}N_2O_2^+$
Exact Mass: 267,1128

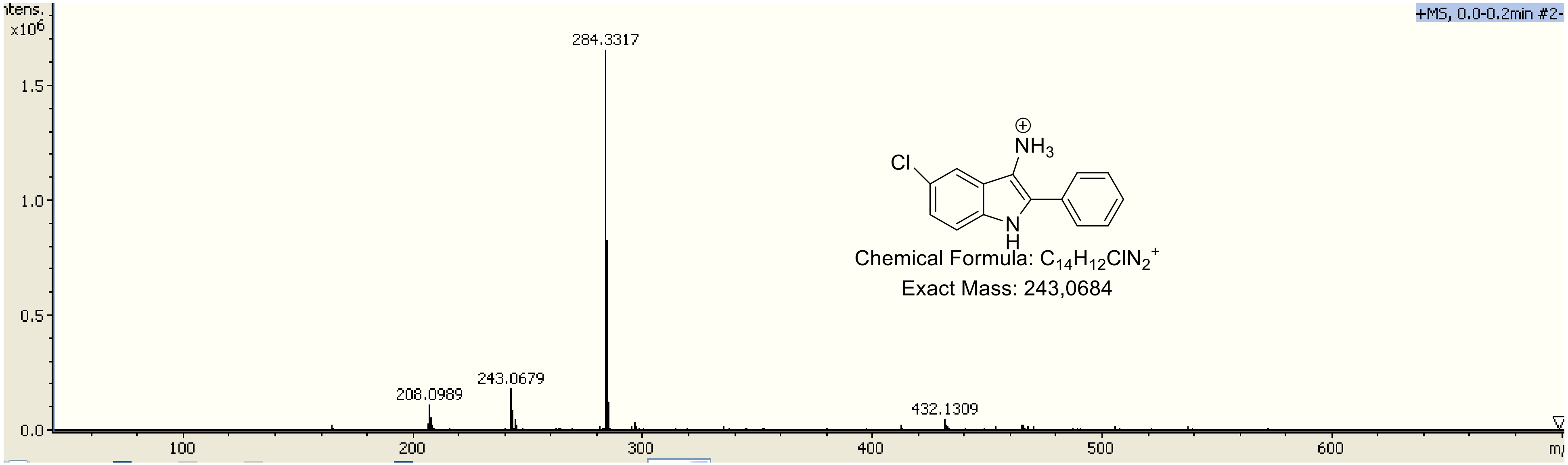
+MS, 0.0-0.2min #2-
x10^6
284.3317
243.0679
208.0989
432.1309
Cl
Chemical Formula: $C_{14}H_{12}ClN_2^+$
Exact Mass: 243,0684

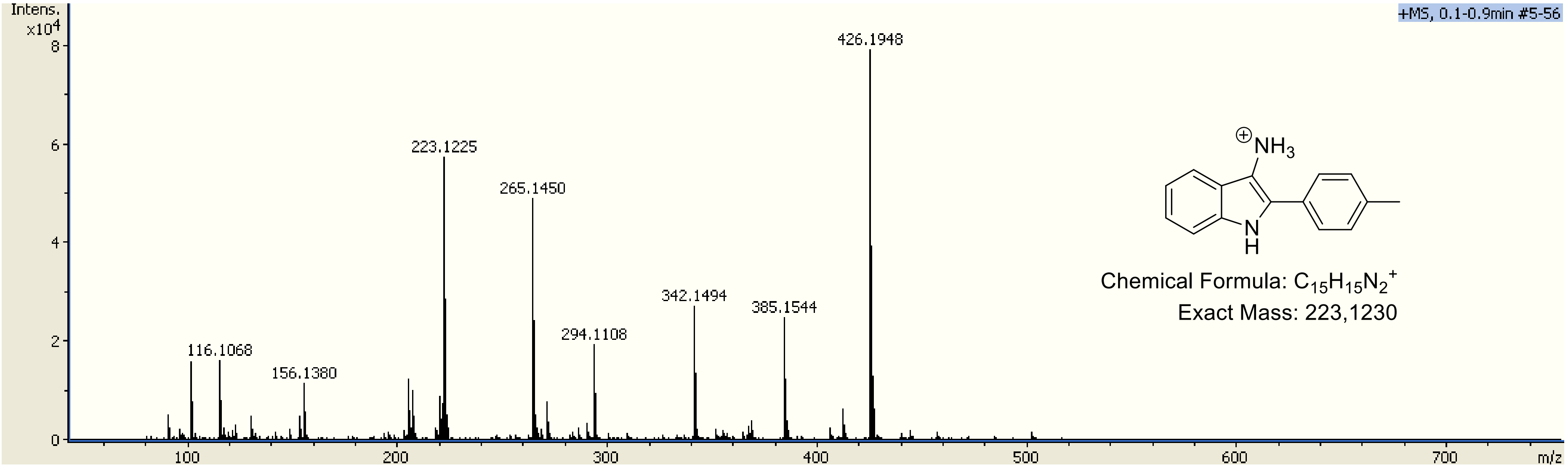

Intens.
x10^4
+MS, 0.1-0.9min #5-56
426.1948
223.1225
265.1450
342.1494
385.1544
294.1108
116.1068
156.1380
m/z
NH3
N
H
Chemical Formula: C15H15N2+
Exact Mass: 223,1230

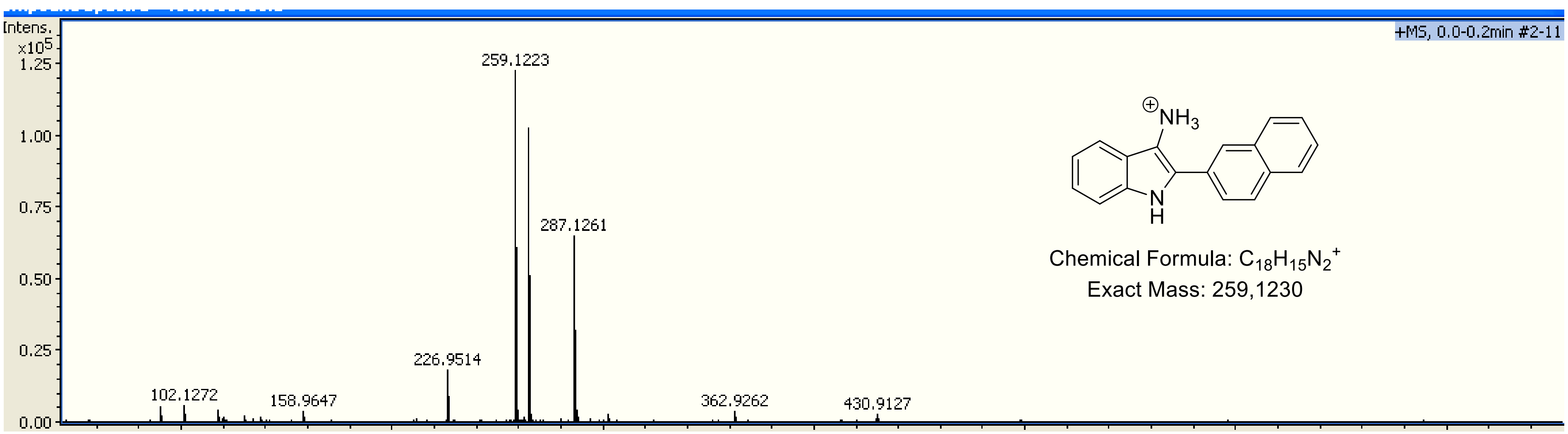

Intens.
x10^5
+MS, 0.0-0.2min #2-11
259.1223
287.1261
226.9514
102.1272
158.9647
362.9262
430.9127
NH3
N
H
Chemical Formula: C18H15N2+
Exact Mass: 259,1230

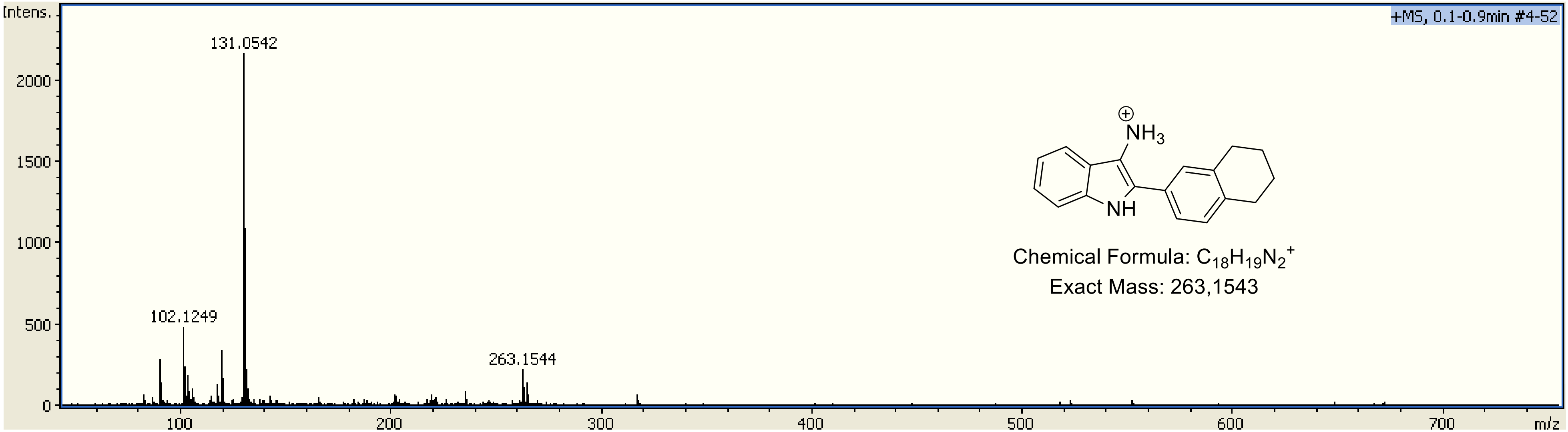
Intens.
+MS, 0.1-0.9min #4-52
131.0542
102.1249
263.1544
Chemical Formula: $C_{18}H_{19}N_2^+$
Exact Mass: 263,1543
m/z

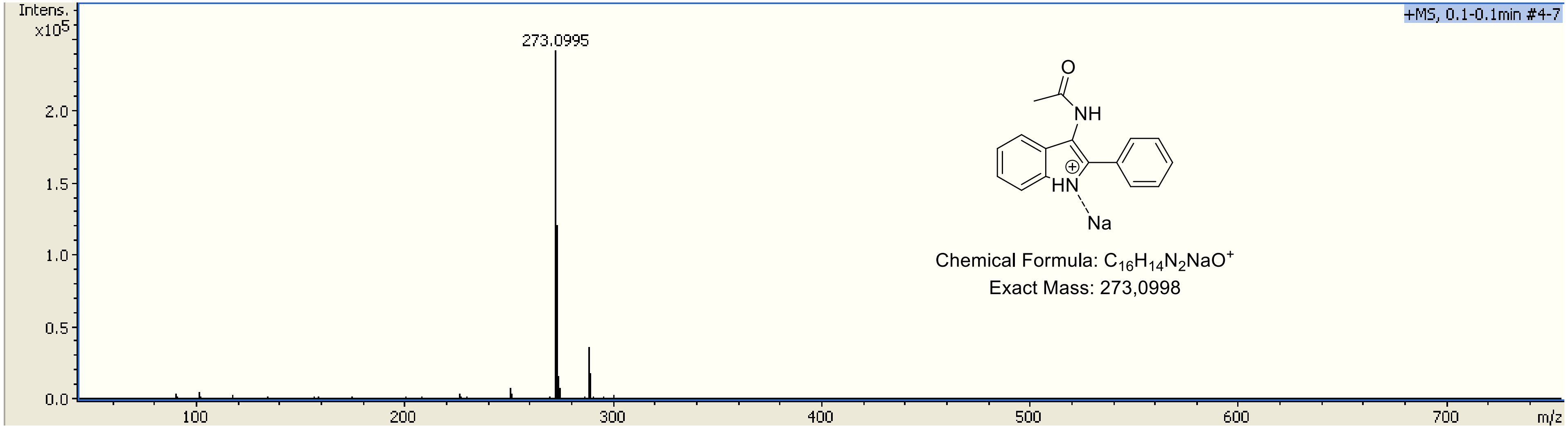
Intens.
x10^5
+MS, 0.1-0.1min #4-7
273.0995
Chemical Formula: $C_{16}H_{14}N_2NaO^+$
Exact Mass: 273,0998
m/z

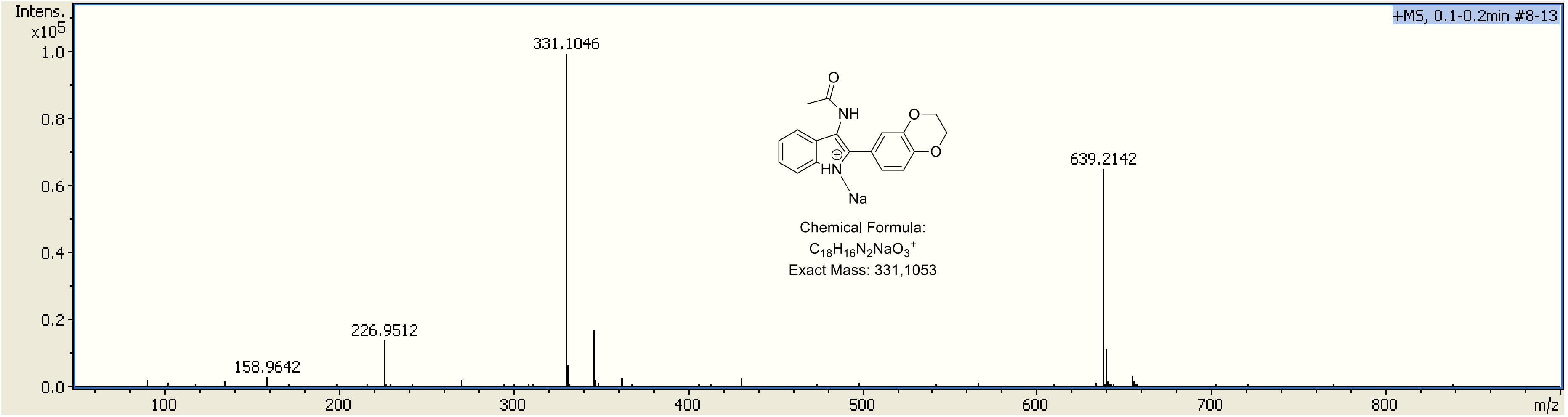
Intens.
x10^5
+MS, 0.1-0.2min #8-13
331.1046
639.2142
226.9512
158.9642
Chemical Formula:
C18H16N2NaO3+
Exact Mass: 331,1053
m/z

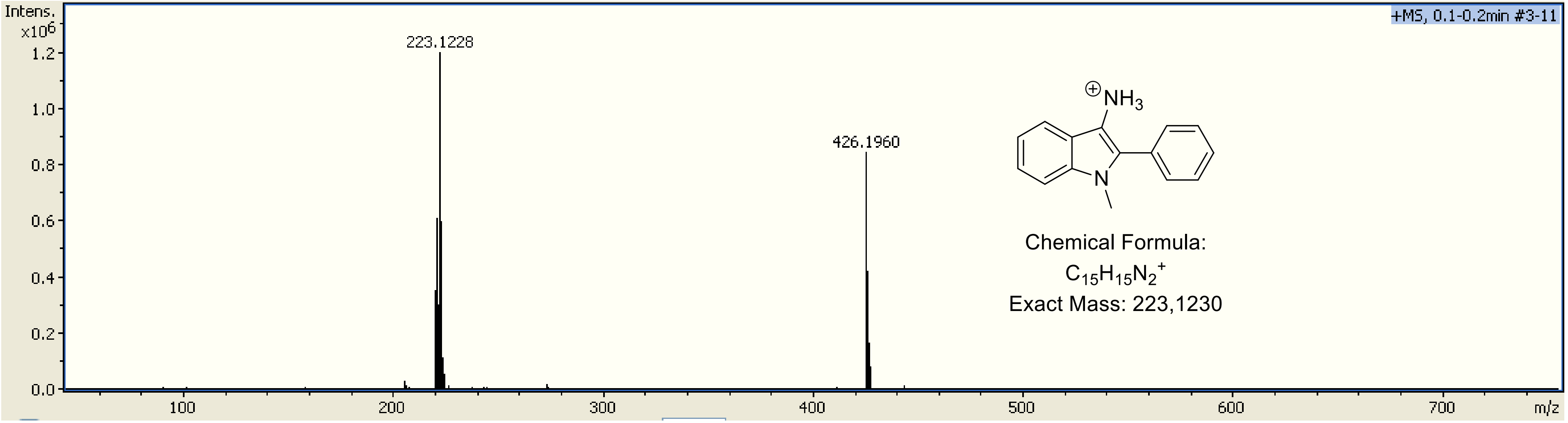
Intens.
x10^6
+MS, 0.1-0.2min #3-11
223.1228
426.1960
Chemical Formula:
C15H15N2+
Exact Mass: 223,1230
m/z
